# JAC1 targets YY1 mediated JWA/p38 MAPK signaling to inhibit proliferation and induce apoptosis in TNBC

**DOI:** 10.1038/s41420-022-00992-9

**Published:** 2022-04-05

**Authors:** Zurong Zhai, Yanlin Ren, Chuanjun Shu, Dongyin Chen, Xia Liu, Yan Liang, Aiping Li, Jianwei Zhou

**Affiliations:** 1 Department of Molecular Cell Biology & Toxicology, Center for Global Health, School of Public Health, Nanjing Medical University, 101 Longmian Avenue, Nanjing, 211166 China; 2 Department of Bioinformatics, School of Biomedical Engineering and Informatics, Nanjing Medical University, 101 Longmian Avenue, Nanjing, 211166 China; 3 Department of Medicinal Chemistry, School of Pharmacy, Nanjing Medical University, Nanjing, 211166 China; 4 Department of Oncology, The First Affiliated Hospital of Nanjing Medical University, 300 Guangzhou Road, Nanjing, 210029 China; 5 Jiangsu Key Lab of Cancer Biomarkers, Prevention and Treatment, Collaborative Innovation Center for Cancer Medicine, Nanjing Medical University, 101 Longmian Avenue, Nanjing, 211166 China; 6 The Key Laboratory of Modern Toxicology, Ministry of Education, School of Public Health, Nanjing Medical University, 101 Longmian Avenue, Nanjing, 211166 China

**Keywords:** Translational research, Breast cancer, Drug development

## Abstract

Triple negative breast cancer (TNBC) is a type of breast cancer with poor prognosis, and has no ideal therapeutic target and ideal medicine. Downregulation of JWA is closely related to the poor overall survival in many cancers including TNBC. In this study, we reported at the first time that JWA gene activating compound 1 (JAC1) inhibited the proliferation of TNBC in vitro and in vivo experimental models. JAC1 specifically bound to YY1 and eliminated its transcriptional inhibition of JWA gene. The rescued JWA induced G1 phase arrest and apoptosis in TNBC cells through the p38 MAPK signaling pathway. JAC1 also promoted ubiquitination and degradation of YY1. In addition, JAC1 disrupted the interaction between YY1 and HSF1, and suppressed the oncogenic role of HSF1 in TNBC through p-Akt signaling pathway. In conclusion, JAC1 suppressed the proliferation of TNBC through the JWA/P38 MAPK signaling and YY1/HSF1/p-Akt signaling. JAC1 maybe a potential therapeutic agent for TNBC.

## Introduction

Breast cancer is the most common cancer [1, 2]. Triple negative breast cancer (TNBC) is a subtype of breast cancer accounting for 15–20% of all breast cancers [3]. The outstanding clinical features of TNBC are the strong metastasis [4] and low survival rate [5]. Unlike ER^+^ and HER2^+^ breast cancers, there are no currently available first-line regimens for TNBC patients [6, 7], mainly relying on conventional chemotherapy [8, 9]. But chemoresistance often occurs gradually [10], which greatly decreasing the clinical efficacy of the drugs [11]. Therefore, it is an urgent unmet need to find new drugs to overcome the current challenges in TNBC therapy.

Yin Yang 1 (YY1) works as a transcription factor and is associated with cell differentiation [12], apoptosis [13], and tumorigenesis [14]. It is reported that the function of YY1 has two sides in different cancers[15]; most researches indicate that YY1 is highly expressed in a range of cancer types including breast [16, 17], gastric, brain, liver, lung, and melanoma [18]. However, YY1 is also reported to play a tumor suppressor role, particularly in the case of pancreatic cancer [19].

JWA, an effective environmental response gene, is also identified as a tumor suppressor gene in cancers [20–22]. Moreover, JWA exerts pro-apoptotic role [23, 24], and most of the anticancer functions of JWA in anti-proliferation and pro-apoptosis are mediated by activating MEK-ERK signaling [25]. Interestingly, JWA promotes As_2_O_3_-induced apoptosis in MCF-7 cells through p38 MAPK signaling [26]. JWA also inhibits the invasion of TNBC cells by down-regulating CXCR4 [20]. These reports demonstrate that JWA plays an important role in suppressing tumors initiation and development.

Recently, we have reported anti-proliferation role of JWA gene agonist JAC1 in HER2 positive breast cancer cells [21]. Herein, we further investigated the effects of JAC1 in TNBC. We identified at the first time that JWA contributes to maintain the homeostasis of cell proliferation and apoptosis and its deficiency might be a new checkpoint in TNBC. More importantly, we determined JAC1 works as a novel effective inhibitor of YY1, it binds to overexpressed YY1 in TNBC cells and removes its transcriptional inhibitory roles on JWA gene, therefore reversed the dysregulation of proliferation/apoptosis signaling in TNBC cells.

## Result

### Downregulation of JWA is associated with poor prognosis in TNBC

**Fig. 1:**
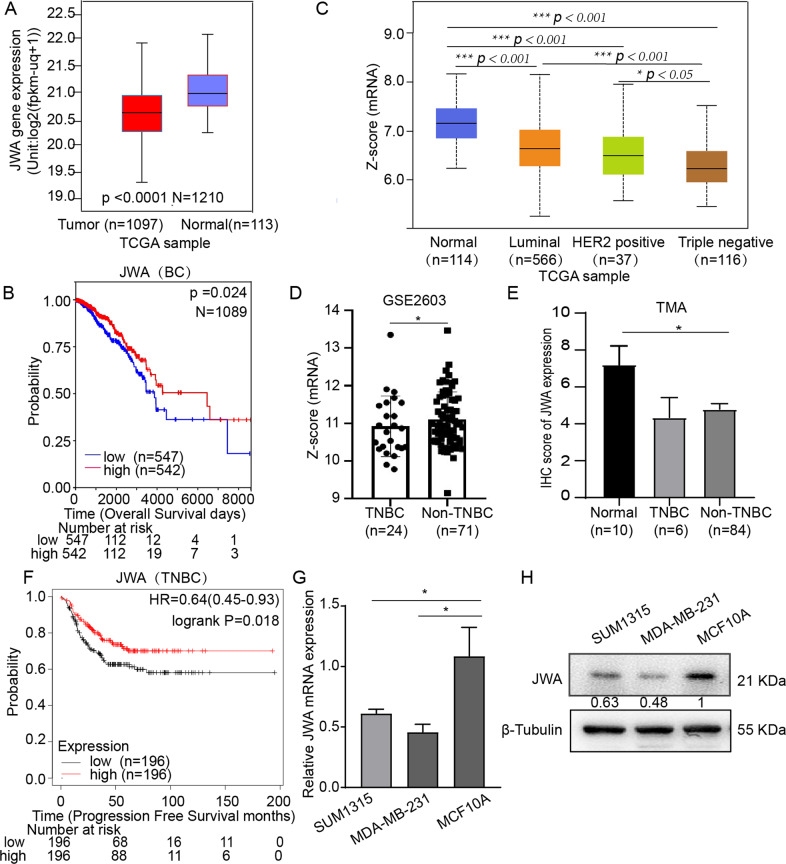
Down regulation of JWA is associated with poor prognosis in TNBC. **A** JWA mRNA expression was interrogated in unpaired cohorts (TCGA database, 1097 cancer samples, 113 non-cancerous samples). **B** Kaplan–Meier curves depicted Overall Survival according to the JWA expression of breast cancer cohort (*n* = 1089). **C** The JWA expression level was analyzed in subtypes of breast cancers. (TCGA database,114 normal; 566 Luminal; 37 HER2 positive; 116 Triple negative). **D** GSE2603 database was analyzed for JWA expression (24 TNBC; 71 non-TNBC). JWA mRNA level was compared between TNBC and non-TNBC. **E** The protein expression of JWA was compared between normal(*n* = 10), TNBC (*n* = 6) and non-TNBC (*n* = 84) tissues. **F** Kaplan–Meier curves depicted progression-free survival according to the expression of JWA in TNBC cohort. *P* values were calculated with the log-rank test. **G** The mRNA expression of JWA in normal breast cell (MCF10A) and TNBC (MDA-MB-231 and SUM 1315) cells (*n* = 3). **H** The protein expression of JWA in normal breast cell (MCF10A) and TNBC (MDA-MB-231 and SUM1315) cells. Z-score = log_2_^(FPKM+1)^. *P < 0.05, **P < 0.01, ***P < 0.001, ****P < 0.0001.

To identify if JWA expression was associated with the prognosis in breast cancer patients, 1210 cancerous and non-cancerous tissues in The Cancer Genome Atlas (TCGA) database were analyzed (https://xenabrowser.net/datapages/). The results showed that JWA was significantly down-regulated in breast cancer tissues (*n* = 1097) compared with adjacent normal breast tissues (*n* = 113) (Fig. 1A). the Overall Survival (OS) of breast cancer patients with high JWA expression is significantly longer than that patients with low JWA expression (https://xenabrowser.net/) (Fig. 1B). Besides, the mRNA expression of JWA was lower in all subtypes of breast cancer than in adjacent normal breast tissues; TNBC tissues had the lowest JWA expression among the subtypes (Fig. 1C). The RNA-seq datasets (GSE2603) from GEO database further confirmed that the mRNA expression of JWA in TNBC tissues was lower than that in non-TNBC tissues (Fig. 1D). In protein level, the expression of JWA was also downregulated in both TNBC and non-TNBC tissues compared with its adjacent normal tissues (*P* < 0.05, Fig. 1E). The results from TCGA database also showed that Progression-Free-Survival (PFS) of TNBC patients was positively correlated with the mRNA expression levels of JWA (https://kmplot.com/analysis/) (*P* < 0.05, Fig. 1F). For further mechanistic investigation of how JWA involves in TNBC development, we detected both the mRNA and protein expressions of JWA in normal breast epithelial cell and TNBC cells. RT-PCR (Fig. 1G) and Western blot (Fig. 1H) results showed that both the mRNA and protein expression levels of JWA were lower in TNBC cells than that in normal breast epithelial cells.

### JAC1 inhibits the proliferation of TNBC cells

**Fig. 2:**
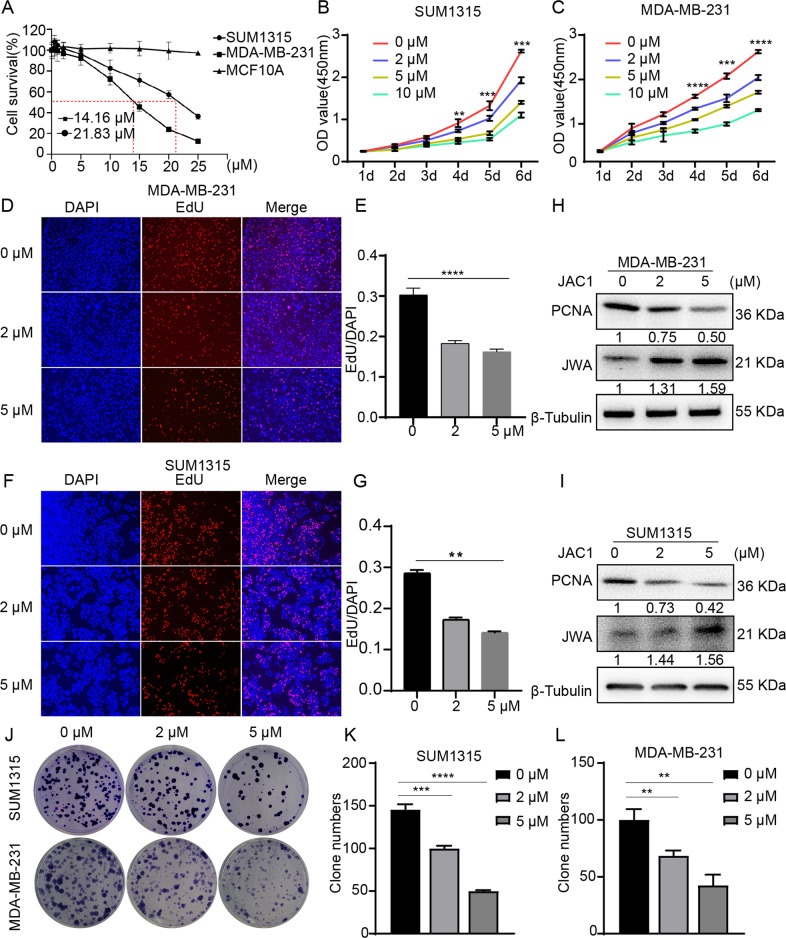
JAC1 inhibits the proliferation of TNBC cells. **A** Viabilities of MDA-MB-231, SUM1315, and MCF10A cells were analyzed by CCK8 after treated by different doses of JAC1 for 24 h (*n* = 5). Viability of SUM1315 cells (**B**) and MDA-MB-231 cells (**C**) were analyzed by CCK8 in 6 days (*n* = 5). **D**, **E** EdU assay of MDA-MB-231 cells was performed to evaluate cell proliferation after treated by different doses of JAC1 for 24 h (magnification, ×50, scale bar, 50 μm), (*n* = 3). **F**, **G** EdU assay of SUM1315 cells was performed to evaluate cell proliferation after treated by different doses of JAC1 for 24 h (magnification, ×50, scale bar, 50 μm), (*n* = 3). The expressions of PCNA protein in MDA-MB-231 (**H**) and SUM1315 (**I**) cells were measured by Western blot after treated with 0, 2, 5 μM JAC1 for 24 h. **J** Colony formation assays were completed for MDA-MB-231 and SUM1315 cells treated by JAC1 (0, 2, 5 μM). The quantitative data of colony numbers of SUM1315 (**K**) and MDA-MB-231 (**L**) cells (*n* = 3). *P < 0.05, **P < 0.01, ***P < 0.001, ****P < 0.0001. N.S. no significant differences.

Given that overexpression of JWA (by transfection of Flag-JWA) suppresses migration and invasion in TNBC cells in vitro [20], and JAC1 inhibits HER2 positive breast cancer cells proliferation by JWA-triggered E3 ubiquitin ligase SMURF1 [21]. To determine whether increased JWA expression inhibited the malignant phenotype of TNBC cells, different doses of JAC1were exposed for 24 h in TNBC cells. The CCK-8 results showed that JAC1 treatment dose-dependently inhibited the cell viability in both MDA-MB-231cells ((IC_50_ = 14.16 μM)) and SUM1315 cells (IC_50_ = 21.83 μM); however, JAC1 did not show significant effect on MCF-10A normal mammary epithelial cells (Fig. 2A). The inhibitory effects of JAC1 on two TNBC cells were also indicated time-response manners (Fig. 2B, C). The EdU incorporating assays showed that the number of EdU-positive cells were reduced dose-dependently after JAC1 treatment for 24 h in TNBC cells (Fig. 2D–G). Western blot assays indicated that JAC1 dose-dependently reduced the expression of PCNA, however, increased JWA expression in TNBC cells (Fig. 2H, I). Similarly, the colony formation assays showed that JAC1 treatment resulted in inhibition of proliferation dose-dependently in TNBC cells (Fig. 2J–L). These data suggested that JAC1 selectively inhibited proliferation in TNBC cells but did not inhibit normal mammary epithelial cells.

### JAC1 triggers apoptosis and G1 phase arrest of TNBC cells

**Fig. 3:**
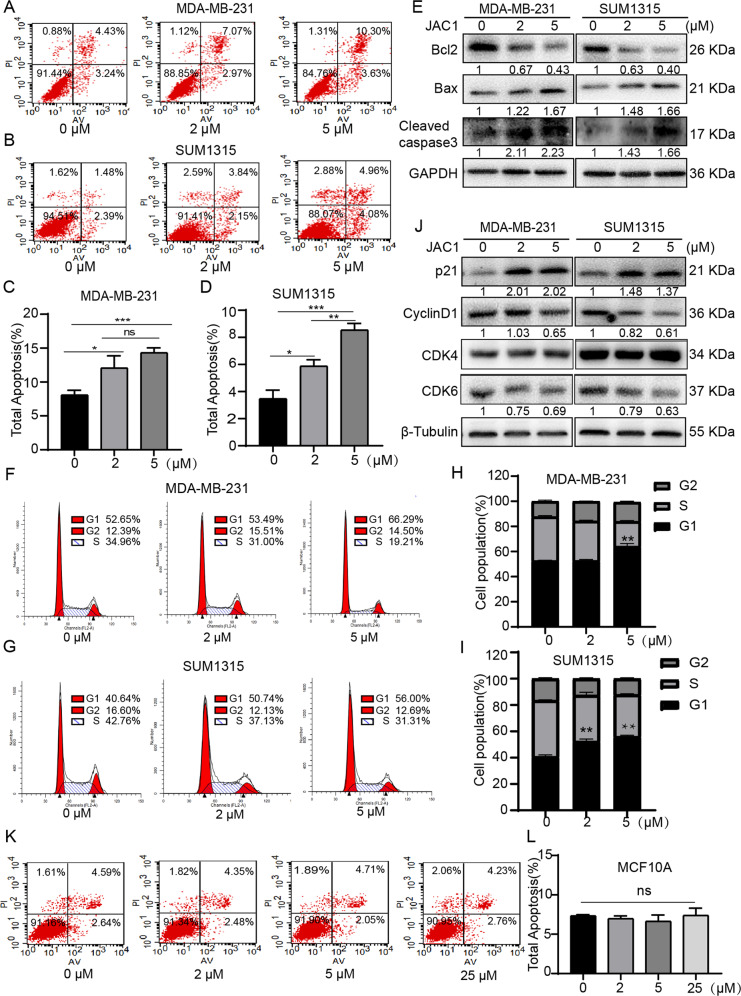
JAC1 triggers apoptosis and cell cycle arrest of TNBC cells. Apoptosis rate was tested using flow cytometry in MDA-MB-231 (**A**) and SUM1315 (**B**) cells after treated with 0, 2, 5 μM JAC1 for 24 h. The statistics of total apoptosis rate in MDA-MB-231 (**C**) and SUM1315 (**D**) cells (*n* = 3). **E** The expressions of apoptosis-related proteins were determined by western blot After TNBC cells were treated with the JAC1 (0, 2, 5 μM) for 24 h. Cell cycle analysis was performed using flow cytometry in MDA-MB-231 (**F**) and SUM1315 (**G**) cells after treated with 0, 2, 5 μM JAC1 for 24 h. The statistics of cell population in MDA-MB-231 (**H**) and SUM1315 (**I**) cells (*n* = 3). **J** The expressions of cell cycle-related proteins were determined by western blot in MDA-MB-231 and SUM1315 cells after treated with 0, 2, 5 μM JAC1 for 24 h. **K** Apoptosis rate was tested in MCF10A cells using flow cytometry after treated with 0, 2, 5, 25 μM JAC1 for 24 h. **L** The statistics of total apoptosis rate in MCF10A cells (*n* = 3). *P < 0.05; **P < 0.01; ***P < 0.001. N.S. no significant differences.

The apoptosis and cell cycle arrest are known key events to cell proliferation [27]. Here we observed the inhibitory effects of JAC1 on cell cycle and apoptosis in the TNBC cell lines. The flow cytometric analysis with Annexin V/PI double staining showed that after treatment with JAC1 for 24 h, the proportion of total apoptotic cells (early and late apoptosis cells) were increased in a dose-dependent manner (Fig. 3A–D). To confirm the proapoptotic effects of JAC1 on TNBC cells, we determined apoptosis-related biomarkers by Western blot in JAC1 treated TNBC cells. The data showed the expression level of Bcl2 was downregulated dose-dependently, at the same time, Bax and cleaved-caspase3 were upregulated after treatment of JAC1 for 24 h (Fig. 3E). The cell cycle assay showed that after treated with 5 μM JAC1 for 24 h, G1 phase cells were increased, however, S phase cells were decreased significantly. (Fig. 3F–I). Meanwhile, the expressions of p21 and CDK6 (not CDK4) were increased but cyclin D1 were decreased significantly (Fig. 3J). Taken together, these results suggested that JAC1 exerted double roles of pro-apoptosis and cell cycle arrest in vitro in TNBC cells. Interestingly, there were no obvious effects on the proportion of total apoptotic cells in JAC1 treated MCF10A (Fig. 3K, L). These results suggest that JAC1 exerted selective inhibitory roles on TNBC cells.

### JAC1 suppresses tumor growth in TNBC xenografted mice

**Fig. 4:**
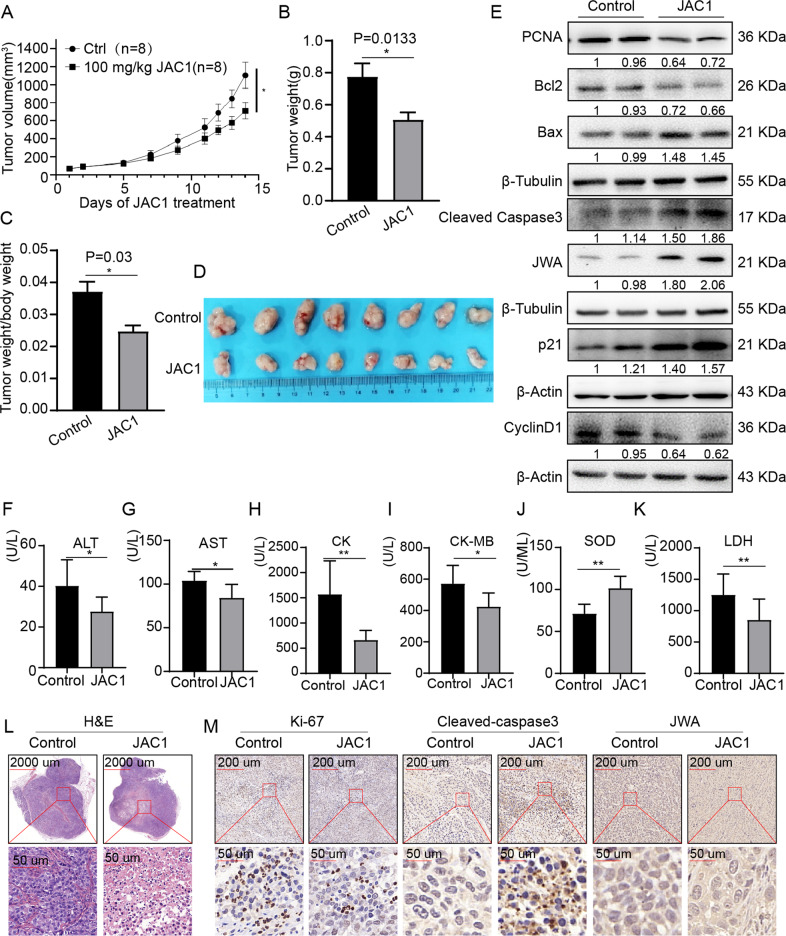
JAC1 suppresses tumor growth in TNBC xenografted mice. **A** The tumor growth curves of MDA-MB-231 cells injection in Control and JAC1 treated groups (*n* = 8). **B** The tumor weight in Control and JAC1-treated groups (*n* = 8). **C** The ratio of tumor weight/body weight in Control and JAC1-treatde groups (*n* = 8). **D** The representative images of subcutaneous tumors in Control and JAC1 treated groups (*n* = 8). **E** Analysis of the expression levels of indicated molecules in tumor tissues of MDA-MB-231 cells tumor-bearing mouse models by Immunoblotting. **F**–**K** The serum biochemical indexes (ALT, AST, CK, CK-MB, SOD, LDH) in control and JAC1-treated mice (*n* = 8). **L** H&E staining of tumor tissues in Control and JAC1 treated groups**. M** IHC staining of Ki-67, Cleaved caspase3, and JWA in Control and JAC1-treated tumor tissues. *P < 0.05; **P < 0.01. N.S. no significant differences.

In order to evaluate the anti-cancer effects of JAC1 in vivo, a xenograft model in nude mouse was constructed. MDA-MB-231 cells (5 × 10^6^ cells) were subcutaneously injected into 5-week-old female BALB/c nude mice. The growth curve of xenografted TNBC tumor in mice was shown in Fig. 4A, the average volumes of tumor were increased more slowly in JAC1 (100 mg/kg/d ×14d) treated mice than those in solvent control mice. At the endpoint of mice model, data confirmed that both tumor weight and the ratio of tumor/body weight were less in JAC1 treated group than in solvent control group; and JAC1 indicated a tumor inhibition rate of 37.4% to TNBC (Fig. 4B–D). Western blot results showed that the expressions of PCNA, Bcl2 and Cyclin D1 were reduced, however, JWA, p21, Bax, and cleaved-caspase3 were increased in JAC1 treated mice tumor tissues (Fig. 4E).

To understand the potential toxic side effects of JAC1 in xenografted mice, we also determined in total of 21 serum biochemical biomarkers. As shown in Fig. 4F–K, JAC1 treatment obviously reduced serum ALT, AST, CK, CK-MB, and LDH, however, increased SOD levels compared to the solvent control treated mice. The other biochemical biomarkers did not show obvious changes by JAC1 treatment (Supplementary Table 2). In addition, H&E staining showed that JAC1 treatment resulted in obvious tissue necrosis in the xenografted tumor mass central area (Fig. 4L); the IHC assay showed Ki67 positive cells were less in JAC1 treated tumor tissue than in control group (Fig. 4M). However, the expressions of cleaved-caspase 3 and JWA were higher in JAC1 treated tumor tissue (Fig. 4M). By the way, no differences were observed in body weight growth curve (Supplementary Fig. 1A) and by H&E staining in liver, heart, spleen, lung, and kidney tissues (Supplementary Fig. 1B) between the two group mice. Moreover, no significant difference was observed between the control group and JAC1-treated group in terms of behavior, feeding pattern, and overall activities. In summary, these results showed that JAC1 effectively inhibited the proliferation of TNBC without significant toxicity both in vivo and in vitro experimental models.

### JAC1 inhibits TNBC progression through JWA mediated activation of p38 MAPK signaling

**Fig. 5:**
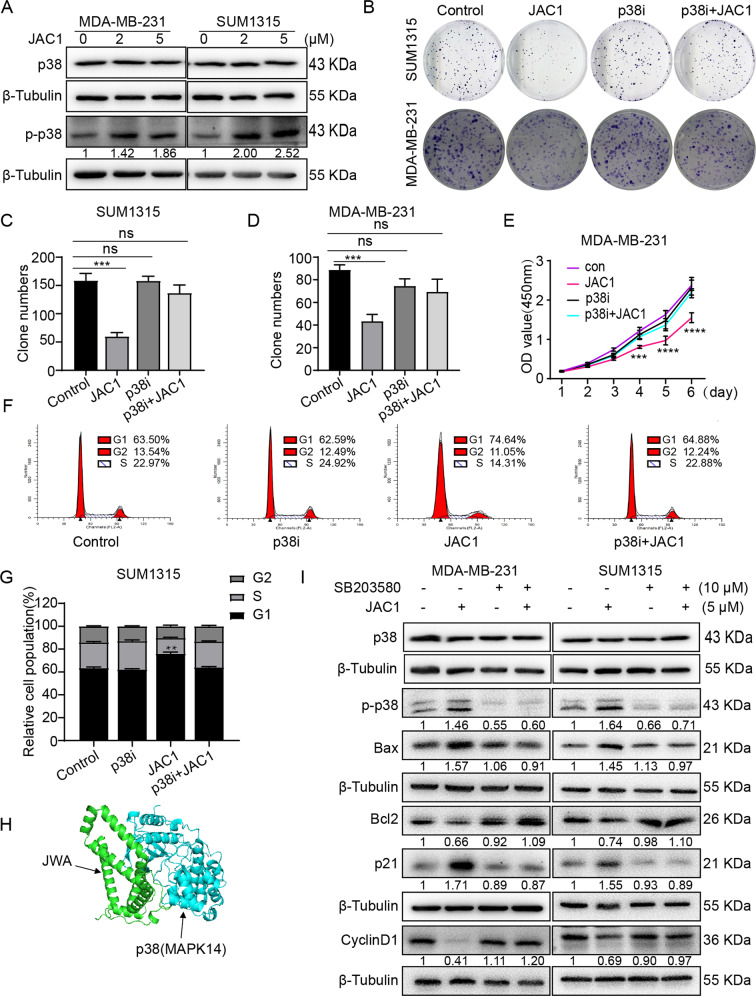
JAC1 inhibits TNBC progression through JWA mediated activation of p38 MAPK signaling. **A** The expressions of p38 and p-p38 were determined by Western blot in both MDA-MB-231 and SUM1315 cells after JAC1 (0, 2, 5 μM) treated 24 h. **B** The inhibition of JAC1 on colony formation via the p38 MAPK pathway was determined in both SUM1315 and MDA-MB-231 cells. The quantitative data of colony numbers in both SUM1315 (**C**) and MDA-MB-231 (**D**) cells (*n* = 3). **E** The proliferation inhibition of JAC1 via the p38 MAPK pathway was determined by CCK-8 assay in MDA-MB-231 cells (*n* = 5). **F** The cell cycle arrest via the p38 MAPK pathway was determined by flow cytometry in MDA-MB-231 cells after treated by 5 μM JAC1 for 24 h. **G** The statistics of relative cell population of MDA-MB-231 cells (*n* = 3). **H** Molecular docking showed the interaction between JWA and p38. **I** The expression levels of indicated molecules (p38, p-p38, Bax, Bcl2, p21, and CyclinD1) were analyzed by western blot in both MDA-MB-231 and SUM1315 cells after treated with JAC1 or SB203580 for 24 h. *P < 0.05; **P < 0.01; ***P < 0.001; ****P < 0.0001. N.S. no significant differences.

Previous studies have shown that overexpression of JWA induces apoptosis in several cancer cells including MCF-7 breast cancer cells by activating p38 phosphorylation [28]. Herein, we determined if the proapoptotic and G1 cell cycle arrest effects of JAC1 were mediated by p38 signal pathway in TNBC. As we predicted, the TNBC cells treated by JAC1 for 24 h induced phosphorylation of p38 with a dose-dependent manner (Fig. 5A), but had no significant effect on p38 expression. To confirm the role of p38-activation in the inhibition of TNBC proliferation by JAC1, p38 inhibitor (SB203580) was used to repeat the colony formation assay in both SUM1315 and MDA-MB-231 cells. Although SB203580 alone treatment did not show effects on colony formation, it reversed inhibitory effects of JAC1 on cell proliferation (Fig. 5B–D). In addition, CCK8 assay also showed that the SB203580 reversed the effect of JAC1 on cell viability (Fig. 5E). Flow cytometric analysis indicated that G1 phase arrest which induced by JAC1 was weakened when co-treatment with JAC1 and SB203580 (Fig. 5F, G). The molecule docking assay further showed the interaction between JWA and p38, and the 180th–182nd amino acids in p38 was one of the interaction regions to JWA (Fig. 5H). Western blot results also confirmed that the expression of p-p38, Bax, p21, Bcl2, Cyclin D1 which induced by JAC1 were reversed by SB203580, however, the expression of p38 was not affected (Fig. 5I). Since the previous studies have shown that JWA exerts anticancer function through MEK/ERK MAPK signal pathway, we also determined whether the p-ERK or p-JNK signaling pathways involved in JAC1 triggered anti-proliferation and pro-apoptosis events. Interestingly, U0126 (the p-ERK inhibitor) intervention only blocked the effects of JAC1 on p-ERK, and failed to reverse the effects of JAC1 on Bax, p21, Bcl2, and Cyclin D1 in both TNBC cell lines (Supplementary Fig. 2A, B). In addition, JAC1 did not show obvious effects on the expression of p-JNK (Supplementary Fig. 2C, D). Taken together, JAC1 triggered G1 phase arrest and pro-apoptotic effects were only dependent on p38 MAPK signaling in TNBC cells.

### JAC1 targets YY1 and removes the transcriptional inhibition of YY1 on JWA in TNBC cells

Given that we recently have identified JAC1 via JWA/p38 signaling mediating Her2 degradation by SMURF1 [21]. Herein, the antagonist role of JAC1 on TNBC cells were also mediated by activation of JWA/p38 signaling. To identify how JAC1 differentially activated JWA in vary breast cancer cells, we completed immunoprecipitation assay and protein sequencing by using biotin-JAC1 treated BT474 breast cancer cells. The data showed biotin-JAC1 enhanced JWA expression (Supplementary Fig. 3A), and six potential transcription factors including YY1, STAT1, p53, CSDE1, LARP7, and ZFX were identified and may be involved in JAC1-induced upregulation of JWA (Supplementary Fig. 3B). To determine which transcription factor(s) worked in target cells, the small interfering RNAs of these six genes were constructed and transfected into cells, separately. As a result, only knocking down YY1 increased the expression of JWA (Supplementary Fig. 3C, D), and the other five factors (CSDE1, LARP7, STAT1, p53, and ZFX) did not show obvious effects on JWA expression (Supplementary Fig. 3E–I).

**Fig. 6:**
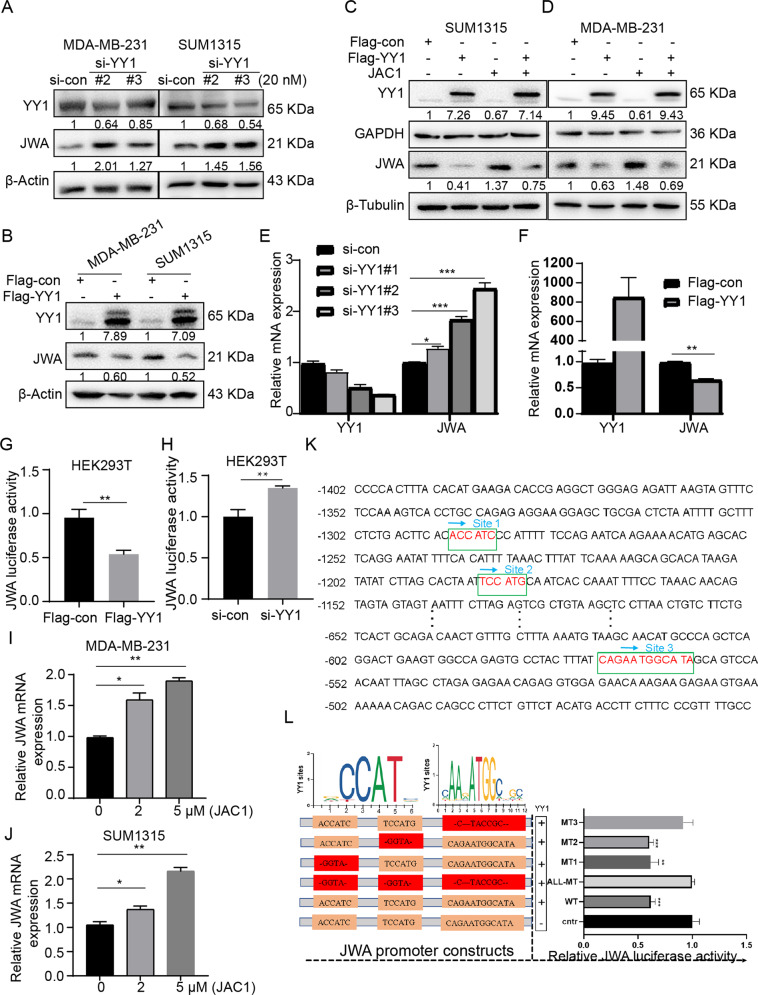
JAC1 targets YY1 and removes the transcriptional inhibition of YY1 on JWA in TNBC cells. The relative expressions of JWA were detected by Western blot after transfected with si-YY1(**A**) or Flag-YY1(**B**) in MDA-MB-231 and SUM1315 cells. JWA expressions were analyzed by Western blot in SUM1315 (**C**) and MDA-MB-231(**D**) cells after treated with JAC1 or Flag-YY1 or co-treated. The JWA mRNA expression was detected by RT-PCR after transfected by si-YY1(**E**) or Flag-YY1(**F**) for 48 h in MDA-MB-231 cells. JWA promoter luciferase activities were analyzed in HEK293T cells after transfected by si-YY1(**G**) or Flag-YY1(**H**) plasmid. **I**, **J** The relative JWA mRNA expression was detected by RT-PCR after treated with indicated doses of JAC1 in MDA-MB-231(**I**) and SUM1315 (**J**) cells. **K** Three putative YY1 binding sites in the JWA promoter regions. **L** JWA promoter Luciferase activities were detected after transfected by luciferase reporter gene plasmid which mutated in putative YY1 binding sites. *P < 0.05; **P < 0.01; ***P < 0.001. N.S. no significant differences.

The computer molecule docking assay also provided supporting evidence and showed an interaction between JAC1 and YY1 and the Lib Dock score is 113.619. The other five candidates were showed lower Lib Dock scores than YY1 (Supplementary Fig. 3J). In TNBC cells, we confirmed that the si-YY1 enhanced JWA protein expressions, and Flag-YY1 inhibited JWA protein expressions (Fig. 6A, B). To verify JAC1 through negatively regulates YY1 to activate JWA expression, Flag-YY1 plasmid was used to treat TNBC cells alone or combined with JAC1. The result indicated that JAC1 could not increase the expression of JWA protein when co-treated with Flag-YY1 plasmid. (Fig. 6C, D). Transfection of si-YY1 into MDA-MB-231 cells increased the mRNA expression level of JWA (Fig. 6E), while transfection of Flag-YY1 decreased the expression level of JWA (Fig. 6F). Luciferase reporter assays showed over-expression of YY1 reduced JWA luciferase activity (Fig. 6G) and down-expression of YY1 increased JWA luciferase activity (Fig. 6H). The dose-response relationship of JAC1 on JWA transcription was also determined in TNBC cells (Fig. 6I, J). To determine YY1 binding motif sequence in JWA gene promoter region, we predicted the potential YY1 binding sites by the promoter analysis tools PROMO (http://alggen.lsi.upc.es/cgi-bin/promo_v3/promo/promoinit.cgi?dirDB=TF_8.3) and JASPAR (http://jaspar.genereg.net/). The results showed three potential YY1 binding motif sequences may be involved in this event (Fig. 6K). Then, we constructed several luciferase reporter gene wild type or mutant plasmids for the responsive JWA promoter region, and conducted assays in HEK293T cells. The results showed only the 3rd motif (−572 to −560 bp; 5′-GAGAATGGCATA-3′) mutant in JWA promoter region lost response to YY1 (Fig. 6L). These results provided further evidence for YY1 as a negative transcription regulator of JWA in TNBC cells.

### JAC1 activates JWA expression by inhibiting YY1 through the ubiquitin–proteasome pathway

**Fig. 7:**
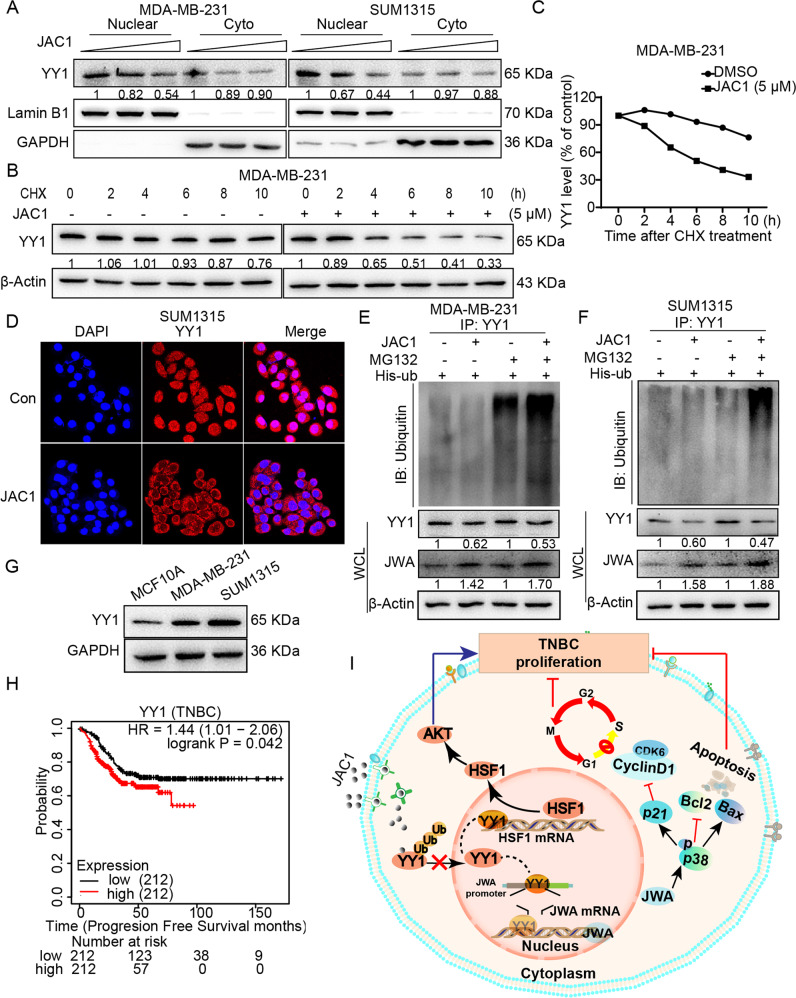
JAC1 activates JWA expression by inhibiting YY1 through the ubiquitin–proteasome pathway. **A** Western bolt assay analyzed the YY1 expression in nucleoplasm and cytoplasm treated with increasing doses of JAC1 in both MDA-MB-231 and SUM1315 cells. **B** YY1 stability assay. MDA-MB-231 cells were treated by JAC1 for 24 h and followed by exposed to CHX for indicated time; expression of YY1 was determined by Western blot. **C** The time-course intensities of the YY1 protein after treated by JAC1 and followed by indicated time of CHX exposed. **D** Immunofluorescence analyzed the expressions and location of YY1 in SUM1315 cells after treated with JAC1 at indicated doses (5 μM). JAC1 increases ubiquitination of YY1. The MDA-MB-231 (**E**) and SUM1315 (**F**) cells were treated with JAC1, followed by MG132; ubiquitination and expression of YY1 were determined by Co-IP and Western blot, respectively. **G** The expression levels of YY1 in MCF10A, MDA-MB-231, and SUM1315 cells. **H** Kaplan–Meier curves depicted survival according to the expression of YY1 in the TNBC cohort from TCGA database. *P* values were calculated with the log-rank test. **I** Schematic diagram of the molecular mechanism of JAC1 inhibiting the proliferation of TNBC.

To elucidate what happened after the interaction between JAC1 and YY1 in TNBC cells, we first observed the intracellular localizations of YY1 after JAC1 treatment. Data showed that JAC1 treatment reduced nuclear YY1 levels in a dose-dependent manner, however, YY1 expression levels were no obvious changes in cytoplasm (Fig. 7A and Supplementary Fig. 4A). The fluorescence staining assay also confirmed that JAC1 treatment significantly reduced nuclear YY1 in SUM1315 cells (Fig. 7D). Then, we performed protein stability assays. The results showed that JAC1 obviously accelerated the degradation of YY1 in MDA-MB-231 cells (Fig. 7B, C). Interestingly, the similar effects of JAC1 on YY1 were relatively weak in SUM1315 (Supplementary Fig. 4B).

We also checked if JAC1 treatment affected mRNA expression of YY1, the data showed there was no obvious effects of JAC1 on transcription of YY1 (Supplementary Fig. 4C, D). In order to confirm whether the degradation of YY1 triggered by JAC1 is mediated by ubiquitin modification, we detected ubiquitin-modified YY1 in His-ub transfected TNBC cells. As shown in (Fig. 7E, F), the ubiquitin levels in JAC1 treated cells were obviously increased. As a result of ubiquitin modification on YY1, the Western blot assay showed JAC1 treatment induced JWA but reduced YY1 expression in TNBC cells. That is to say, JAC1 treatment caused ubiquitination and degradation of YY1 protein, removed its negative transcriptional regulation on JWA, thereby increased JWA expression levels.

### JAC1 regulates the expression of YY1 downstream genes

The YY1 has been identified extensively overexpressed in malignant tumors including breast cancer. The expression of YY1 was higher in TNBC cells than in normal cells (Fig. 7G). TCGA database also showed that breast cancer patients (Supplementary Fig. 5A) or TNBC patients (Fig. 7H) with high YY1 expression had shorter survival than those with low YY1 expression ones.

Given that YY1 activates the expression of HSF1 and promotes the proliferation of TNBC through the Akt signaling pathway [29]. We also verified this signaling in JAC1 treated TNBC cells. Data showed HSF1 mRNA expression was increased by transfection of YY1 in TNBC cells (Supplementary Fig. B, C). Furthermore, YY1-HSF1-p-Akt signaling was also confirmed in JAC1 treated TNBC cells (Supplementary Fig. 5D). These results suggested that as a small molecule compound targeted to YY1, the tumor suppressive effects of JAC1 on TNBC includes at least the following two paths: on the one hand, it released the effect of downstream tumor suppressive target genes such as JWA; On the other hand, it reduced the role of downstream oncogenes such as HSF1. Based on these above evidences, here we summarized the mode of action, how JAC1 worked as a potential TNBC inhibitory agent (Fig. 7I).

## Discussion

For decades, TNBC has been always a clinical problem because there is no targeted therapy drug [30]. This study was designed to address this unmet clinical need. Our data clearly indicated that the anti-proliferation and pro-apoptosis effects of JAC1 on TNBC were triggered through both released tumor suppressor JWA and disrupted the interactions between YY1 and HSF1. YY1 was determined as the direct target of JAC1. The mechanistic evidences showed that both G1 phase arrest and the re-balance of Bcl2/Bax in TNBC were the action mode of JAC1 driving JWA/p38 MAPK signaling. In addition, JAC1 targeting YY1 also blocked the YY1-HSF1 interaction and consequent YY1/HSF1/Akt pathway in TNBC cells.

The p38 MAPK is a stress-activated protein kinase, which is mainly activated by multiple extracellular stresses and inflammatory cytokines [31, 32]. Previous studies have shown that activated p38 MAPK leads to cell cycle arrest and apoptosis [33]. Consistent with this, the phosphorylation of p38 induced by JAC1 was associated with the expression levels of p21, Cyclin D1, Bax, Bcl2, and activated caspase-3. Cell proliferation is primarily controlled by several checkpoints of cell cycle [34]. In the present study, JAC1 arrested the cell cycle at G1 phase in a dose-dependent manner by increasing p21 through p38 MAPK signaling pathway.

YY1 is widely expressed in mammalian cells [35], and associated with poor clinical outcomes [18, 36], involving in cellular differentiation [12], DNA repair [37], cell survival [38] vs. apoptosis [13], etc. However, as a two sides transcriptional factor, the mechanism of YY1 is not completely determined [39]. Besides, even in breast cancer, YY1 has been reported with two-side effects (cancer suppression/cancer progression) [16, 40]. In this study, YY1 inhibited the transcription of JWA, which may be related to the part of YY1’s DNA binding to zinc fingers [35], and the detailed evidences are need to be further clarified.

Ubiquitination is one of the mechanisms which cells maintain homeostasis of related protein expression levels [21]. Instigated by E3 ligases, ubiquitylation controls many processes that are fundamental for development, such as cell division, fate specification, and migration [41, 42]. In this study, JAC1-YY1 interaction accelerated YY1 ubiquitin modification and degradation. However, the behind mechanisms which JAC1 induced YY1 ubiquitination still needs to clarify. Although the E3 ubiquitin ligase Smurf2 is reported to target and degrade YY1 protein in several cancers [43], unfortunately, we did not obtain positive evidence of Smurf2 on YY1 in TNBC.

In summary, in this study, we determined at the first time that JAC1 is a YY1 targeting compound in TNBC cells. The inhibitory effects of JAC1 on TNBC were due to its binding of YY1 therefore rescued anti-cancer functions of JWA gene via both G1 phase arrest and pro-apoptosis. In addition, the binding of JAC1 to YY1 also promoted ubiquitination-mediated degradation of YY1, therefore, removed the oncogenic functions of HSF1 in TNBC. Further investigations are warranted to clarify how the JAC1-YY1 interaction triggers YY1 ubiquitination and degradation, and whether the anti-proliferation and pro-apoptotic effects of JAC1 on TNBC are suitable for other types of cancer.

## Materials and methods

### Chemicals and reagents

JAC1 and Biotin-JAC1 were synthesized by the laboratory-self, with the purity of > 98%. Primary antibodies information is listed in Supplementary Table 1. ECL advance reagent, CCK-8 Kit (C0038), Nucleoplasmic protein isolation Kit (P0027), Luciferase reporter gene Kit (RG027), Protein A/G Plus-Agarose (P2055) were purchased from Beyotime (Shanghai, China). Annexin V-FITC/PI apoptosis detection Kit (A211-02), HiScript III RT SuperMix for qPCR (+gDNA wiper) (R323-01), AceQ qPCR SYBR Green Master Mix (without ROX) were obtained from Vazyme Biotech Co (Nanjing, China). CHX (761982), MG132 (C2211) were obtained from Sigma (St. Louis, MO).

### Cell culture

Human breast cancer cells (SUM1315, MDA-MB-231, and BT474) and human embryonic kidney cells HEK 293T were purchased from the American Type Culture Collection (ATCC; Manassas, VA, USA) and all the cells were maintained in Dulbecco’s modified Eagle’s medium (DMEM) containing 10% or 20% fetal bovine serum (FBS; Gibco, Grand Island, NY, USA) and 1% penicillin–streptomycin (HyClone, Logan, UT, USA). The human normal breast epithelial cells MCF-10A were purchased from Zhong Qiao Xin Zhou and cultured in a special medium (5% horse serum, 20 ng/mL epidermal growth factor, 0.5 µg/mL hydrocortisone and antibiotic-antimycotics, 0.1% penicillin, streptomycin). All the cells were incubated at 37 °C in a 5% CO_2_ humidified atmosphere.

### Western blot assays

Cell proteins were lysed with RIPA buffer (50 mM Tris, pH 7.4; 150 mM NaCl; 1% NP-40; 0.5% sodium deoxycholate; 0.1% SDS; and the 1% protease inhibitor) for 30 min, 4 °C. The xenograft tumor tissue proteins were prepared in tissue protein extraction reagent (Thermo Fisher Scientific). The protein (40 μg/20 μL) was separated equal amounts of by SDS-PAGE and transferred to PVDF membrane. Then the membrane was blocked for 1 h using 5% skim milk at room temperature, and the primary antibodies were used and incubated at 4 °C overnight. After washing 5 times with PBST (10 min/time), the membranes were incubated with peroxidase-conjugated anti-rabbit IgG or anti-mouse IgG (1:1000 diluted, Beyotime, Shanghai, China) for 60 min. The blot images were obtained from multifunctional gel imaging system (Bio-Rad, USA).

### Colony formation assay

In total of 800 cells for both SUM1315 or MDA-MB-231 were made into single-cell suspension and seeded in six-well plates and then cultured in DMEM containing 10% FBS for 10–14 days. The fresh medium containing the corresponding dose of JAC1 (or p38 inhibitor) was updated every 72 h. About two weeks later, the cell culture dishes with colonies were fixed with 4% paraformaldehyde for 30 min, stained with 0.1% crystal violet for 2 h at room temperature, and then photographed.

### CCK-8 assay

The SUM1315, MDA-MB-231, and MCF-10A cells were seeded at the density of 3 × 10^3^ cells/well/100 μL into 96-well plates and were maintained in an incubator at 37 °C and 5% CO_2_. Then CCK8 reagent (10 μL) was added to each well and incubated for 2 h. The absorbance at 450 nm (A450) was measured on a microplate reader and calculated the cell survival ratios. This study conducted at least three replicate holes under each condition.

### Cell apoptosis assay

The cells were seeded at the density of 1 × 10^5^ cells/well/2 mL in six-well plate and then collected and washed with cold PBS for 3 times at the endpoint of culture. After centrifugation, the cell pellet was suspended with 100 μL of binding buffer; 5 μL Annexin V and 5 μL PI were added and incubated for 15 min in dark at room temperature according to the manufacturer’s protocol. Then, flow cytometry (BD Biosciences, NJ) was used to detect apoptotic cells within 1 h. Each experiment had three replicates.

### Cell cycle analysis

The cells were seeded at the density of 1 × 10^6^ cells/well/2 mL in six-well plate and collected and washed 3 times with PBS at the endpoint of culture, and then fixed with ice-cold 70% ethanol for at least 18 h. Centrifugation and washing with PBS, then PI staining solution was added and incubated for 15 min in dark at room temperature. The cell cycle was analyzed using flow cytometry (BD Biosciences, NJ). Each experiment had 3 replicates.

### Co-immunoprecipitation assay (CO-IP)

The cells were firstly washed twice with PBS at the end point of culture, then collected and lysed with IP lysis buffer at 4 °C for 30 min, and centrifuged for 15 min at 4 °C, 12,000 × *g*. The supernatant was transferred to a new centrifuge tube, added the anti-biotin primary antibody to 500 μg total protein and incubated overnight at 4 °C. The negative control group was added with the IgG (mouse IgG or rabbit IgG). 20 μL of protein A/G was added in all samples and co-incubated overnight at 4 °C. The samples were washed with ice-cold PBS for 4 times, then add 60 μL 2 × SDS loading buffer to agarose magnetic beads precipitation and boiled at 100 °C for 5 min. Then the protein expressions were detected by Western blot.

### Immunofluorescence (IF) assay

The cultured cells were removed medium at first and fixed with methanol for 30 min, further added in 10% normal goat serum for 1 h to block non-specific binding sites at room temperature. YY1 antibody was then used to incubate cells for overnight at 4 °C; the CY3 labeled fluorescent secondary antibody was incubated the cells for 2 h at room temperature, and then the nucleophilic dye DAPI was stained the nucleus for 15 min. Zeiss AIM software was used to capture confocal images of the cells on a Zeiss LSM 700 confocal microscope system (Carl Zeiss Jena, Oberkochen, Germany).

### Quantitative real-time PCR analysis

Total RNA was isolated with Trizol reagent (Invitrogen, Carlsbad, California, USA). cDNA was synthesized from 1 μg of total RNA using the Hiscript Q RT Super Mix regent according to the manufacturer’s protocol. The quantitative RT-qPCR was performed using ABI Prism 7900 Sequence detection system (Applied Biosystems, Canada) and SYBR Green PCR Master Mix (TaKaRa Bio, Japan). Thermal cycling conditions of the PCR were 5 min at 94 °C, 36 cycles of 35 s at 94 °C, 30 s at 56 °C, and 35 s at 72 °C. The expression levels of mRNA were defined based on the threshold cycle (Ct), and relative expression levels were calculated using the 2^−ΔΔCt^ method. The expression level of GAPDH mRNA was used as reference genes.

The respective primer sequences were:

JWA: 5′-TCTGAGGTCTTCTCTCTGAAACATC-3′ and 5′-CTTCGGAACCTCAAGAACAAAC-3′ HSF1: 5′- AACACAGCCCCTATGGACA −3′ and 5′-GTCTGCAGGTTGTCCAGGTT-3′ YY1: 5′-TACCTGGCATTGACCTCTC-3′ and 5′-GGCCGAGTTATCCCTGA-3′

GAPDH: 5′-AATGAAGGGGTCATTGATGG-3′ and 5′-AAGGTGAAGGTCGGAGTCAA-3′

### Plasmids and small interfering RNA (siRNA) transfection

Human specific siRNA was synthesized by GENERAY (shanghai, China), and the plasmid pcDNA3.0-Flag-YY1 was synthesized by You Bao (Shanghai, China). The transfection assay was performed using Lipofectamine™ 3000 Reagent in Opti-MEM medium (Invitrogen, Carlsbad, CA, USA) according to the manufacturer’s protocols. After 48 h of transfection, the cells were harvested for RT-PCR or Western blot assays.

The siRNA sequences were:

si-YY1:

1. 5′-CCUCCUGAUUAUUCAGAAUTT-3′and 5′-AUUCUGAAUAAUCAGGAGGTT-3′;

2. 5′-CCAAACAACUGGCAGAAUUTT-3′and 5′-AAUUCUGCCAGUUGUUUGGTT-3′;

3. 5′-GCUCCAAGAACAAUAGCUUTT-3′and 5′-AAGCUAUUGUUCUUGGAGCTT-3′;

si-CSDE1:

1. 5′-GGACAGAAAUGGUAAAGAATT-3′and 5′-UUCUUUACCAUUUCUGUCCTT-3′;

2. 5′-GGGCACGGUUUCAUUUCAUTT-3′and 5′-AUGAAAUGAAACCGUGCCCTT-3′;

3. 5′-GCCAAGGAUGUGGAAGGAUTT-3′and 5′-AUCCUUCCACAUCCUUGGCTT-3′;

sh-p53:

1. GTACCACCATCCACTACAA;

2. AGAGAATCTCCGCAAGAAA;

3. GGAGTATTTGGATGACAGA;

sh-LARP7:

1. GGAGAAAGTTAATGCAACA;

2. GCTGGAAACTCGAGATCCT;

3. GCGAATGGATGGATTTGAA;

sh-ZFX:

1. GCAAATGGATGACAATGAA;

2. GTCGGAAATTGATCCTTGT;

3. TGCTGAAATCGCTGACGAA;

sh-STAT1:

1. CTGGATATATCAAGACTGA;

2. GCACGCTGCCAATGATGTT;

3. CATGCGGTTGAACCCTACA;

### Luciferase reporter gene assay

HEK 293T cells, 5 × 10^4^ well/500 μL, were seeded and cultured overnight in 24-well plate. Then the cells were transfected with either wild-type or mutant reporter gene plasmid using Lipo3000 reagent according to the manufacturer’s instructions. After 48 h, the cell lysates were collected and the activities of firefly and Renilla luciferases were measured by a Dual-Luciferase Reporter System (Promega). The dual luciferase kit (Beyotime, RG027) was used to measure luciferase activity. Relative luciferase activity was standardized by the Renilla luciferase. Each experiment was repeated three times.

### Ubiquitination assay

Both SUM1315 and MDA-MB-231 cells transiently transfected with Ubiquitin (Ub) plasmid for 48 h were incubated with 5 μM JAC1 for 24 h and then treated with MG132 (10 μM) for another 6 h. Extracted whole cell lysates protein was incubated with anti-YY1 antibody for 24 h at 4 °C. 20 μL Protein A/G Plus-Agarose beads were added and shaken overnight. And then the beads were washed with pre-cooled PBS for 4 times, and centrifuged at 1000 × *g* for 5 min at 4 °C; the IP product was then detected by Western blot.

### Protein structure analysis

Three-dimensional structures of JWA and p38 or JAC1 and Transcription factors, such as YY1, CSDE1, ZFX, LARP7, STAT1, and P53 were predicted using the Alpha Fold protein structure database (https://alpgafold.ebi.ac.uk/). The largest possible interaction region of proteins was then obtained by Discovery Studio 3.0, respectively. These predicted binding pockets were utilized to construct an initial coarse model of the complex. Then, the complex was optimized by Rosetta software. The optimization model for complex was then obtained based on energy scores. Binding sites between proteins in complex were then obtained by RING (residue interaction network generator) software. PyMol software was utilized to show High-quality 3D images of structures.

### Xenograft tumor mice model

The mice model was pre-approved by the Ethics Committee of Nanjing Medical University (IACUC-1811067). 5-week-old female BALB/c mice were purchased from Model Animal Research Center of Nanjing University (Nanjing, China) and maintained in SPF facilities. MDA-MB-231 cells (5 × 10^6^) were inoculated subcutaneously into the right axilla of each nude mouse, respectively. When the tumor volume reached in average 60–100 mm^3^ (tumor volume V = 0.5 × length × width^2^), the mice were randomly divided into different groups for further experimental manipulations (*n* = 8 per group). JAC1 was administered daily by oral gavage according to the mouse body weight (100 mg/kg/mouse; the solvent composition contained 50% saline, 47.5% PEG40, and 2.5% absolute ethanol). The mice body weight and tumor volume were measured every two days. At the end of the experiment, the mice were euthanized, and blood was collected to measure the serum biochemical indicators. The tumor tissues were collected either by formaldehyde fixation or −80 °C cryopreservation for further analysis.

### Statistical analysis

All data were expressed as mean ± standard deviation (SD) and analyzed by SPSS 23.0 (SPSS Inc., Chicago, IL, USA) and GraphPad Prism 8.0 software. Use Student’s t-test to determine the statistical difference between the two groups, while use one-way analysis of variance to determine the statistical difference between multiple groups. *P* < 0.05 was considered to be statistically significant. (*P < 0.05; **P < 0.01; ***P < 0.001). All data were the results of three parallels.

## Supplementary information

Supplemental Figures and Tables

Original Data File

## Data Availability

The data that were analyzed during the current study are available from the corresponding author on reasonable request.
