# Supplementary material for: JAC1 targets YY1 mediated JWA/p38 MAPK signaling to inhibit proliferation and induce apoptosis in TNBC
Source: Cell Death Discov. 2022 Apr 5;8:169. doi: 10.1038/s41420-022-00992-9 (PMC8983694; doi:10.1038/s41420-022-00992-9)
Supplement: Supplementary file 1 — Supplemental Figures and Tables [file 41420_2022_992_MOESM1_ESM.pdf]

Supplemental Figures

Figure S1

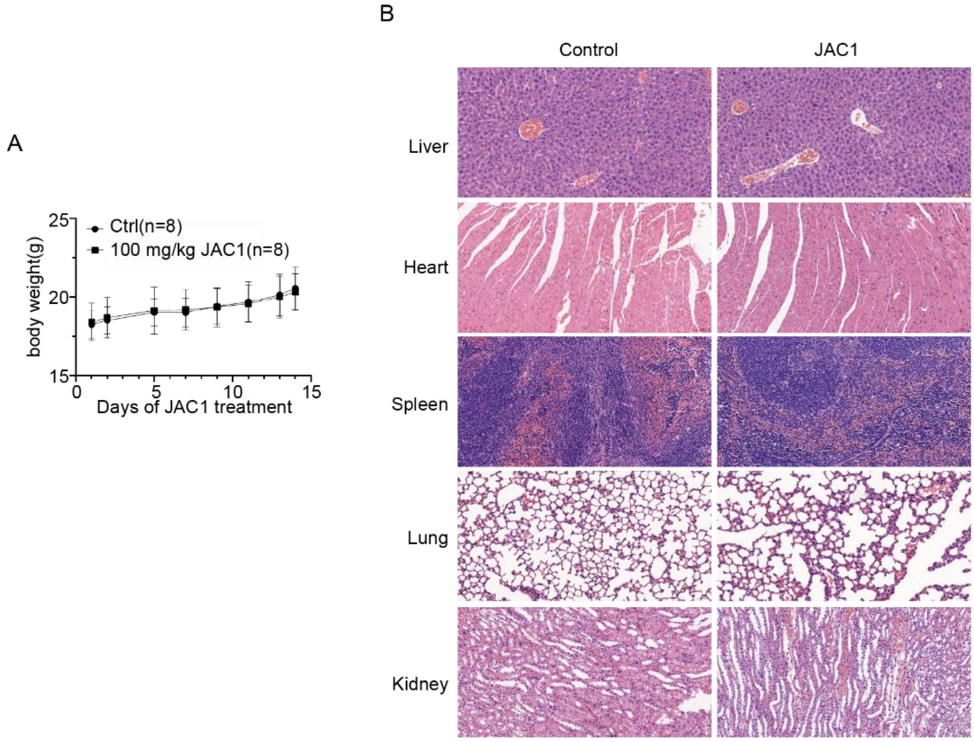

Supplementary Fig. 1 Mouse body weight curve and H&E staining of organs.

A The mouse body weight curves of MDA-MB-231 cells injection in Control and JAC1 treated groups (n=8). **B** H&E staining of major organs (liver, heart, spleen, lung, kidney).

Figure S2

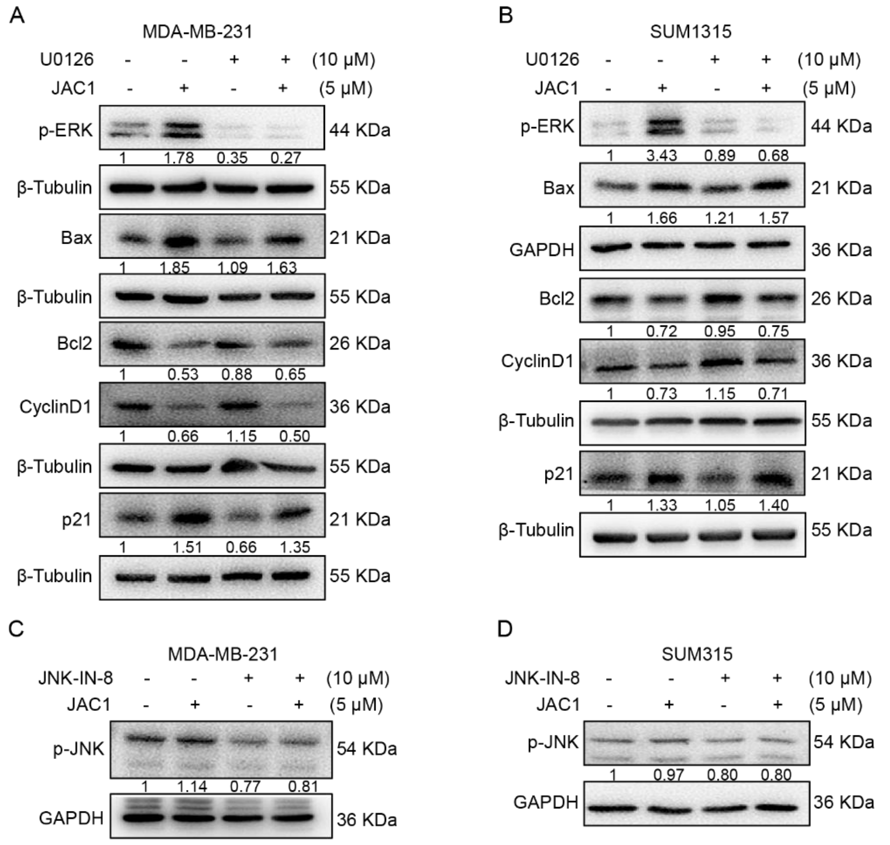

Supplementary Fig. 2 The influence of JAC1 on ERK and JNK signaling pathway.

A-B The expression of indicated molecules treated by ERK inhibitor(U0126) was analyzed by Western blot in both MDA-MB-231(**A**) and SUM1315(**B**) cells. **C-D** The effect of JAC1 treatment on JNK activity in both MDA-MB-231(**C**) and SUM1315(**D**) cells.

Figure S3

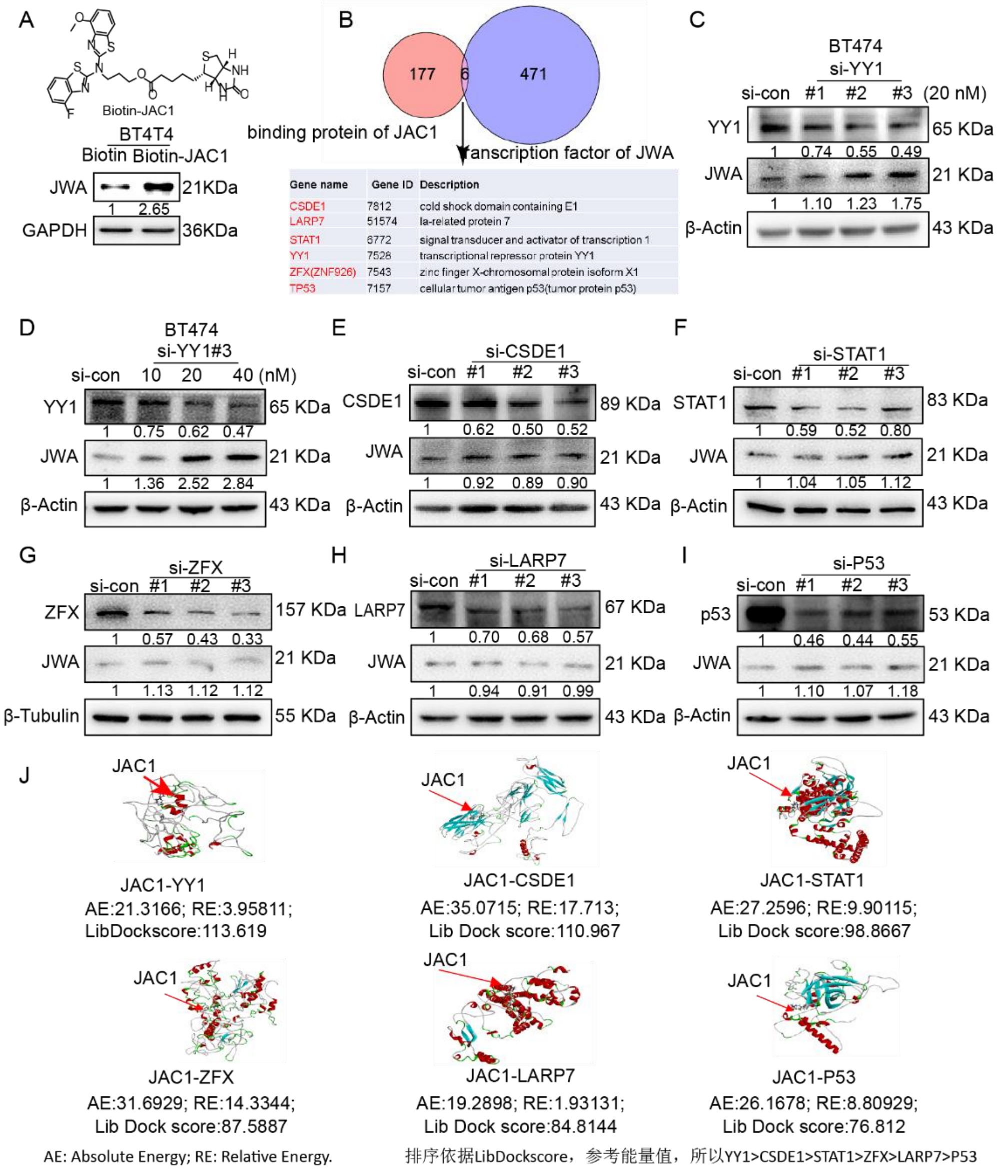

Supplementary Fig. 3 JAC1 interacts with transcription factor YY1 to stimulate JWA expression in BT474 cells.

A The chemical structure of Biotin-JAC1 (up), the expression of JWA (down) was detected by Western blotting after treating BT474 cells with 10μM Biotin-JAC1 for 24 h. **B** Comparison of JAC1 binding proteins and transcription factors of JWA. Numbers in Venn diagram represent the number of JAC1 binding proteins and transcription factors of JWA. **C** The expression of JWA was detected by Western blot after transfected with si-YY1 in BT474 cells. **D** The effect of indicated doses of si-YY1 transfection on JWA expression level. **E-I** The protein expressions of JWA were

detected after transfection with si-CSDE1, si-LARP7, si-STAT1, si-p53 or si-ZFX in BT474 cells, respectively. **J** The proposed binding mode and binding force of JAC1 and YY1, CSDE1, LARP7, STAT1, p53, ZFX were showed.

Figure S4

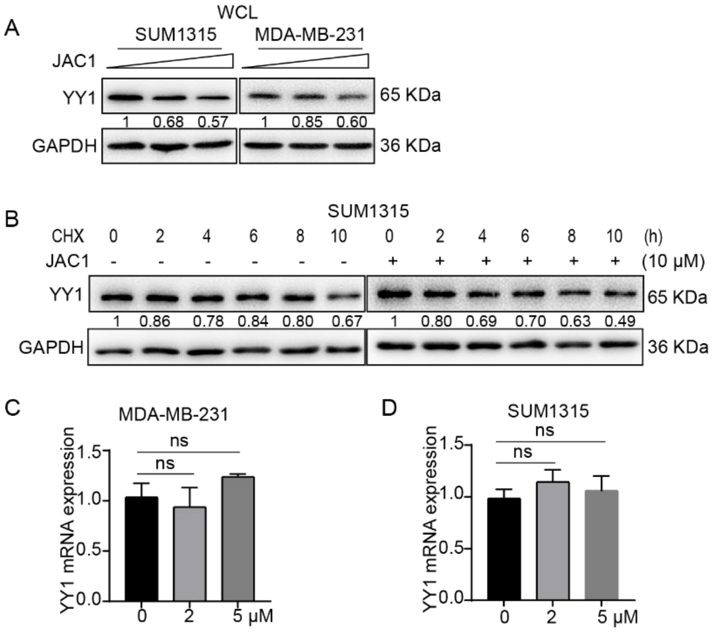

Supplementary Fig. 4 JAC1 reduces YY1 levels at the protein level.

A YY1 expressions in whole cell lysis were detected after treated with increasing doses of JAC1 in both MDA-MB-231 and SUM1315 cells. **B** YY1 stability assay. SUM1315 cells were treated by JAC1 for 24 h and followed by exposed to CHX for indicated time; expression of YY1 was determined by Western blot. **C-D** The mRNA expression of YY1 was detected by RT-PCR in both MDA-MB-231(**C**) and SUM1315 (**D**)cells after treated by JAC1 for 48h (n=3). N.S. no significant differences.

Figure S5

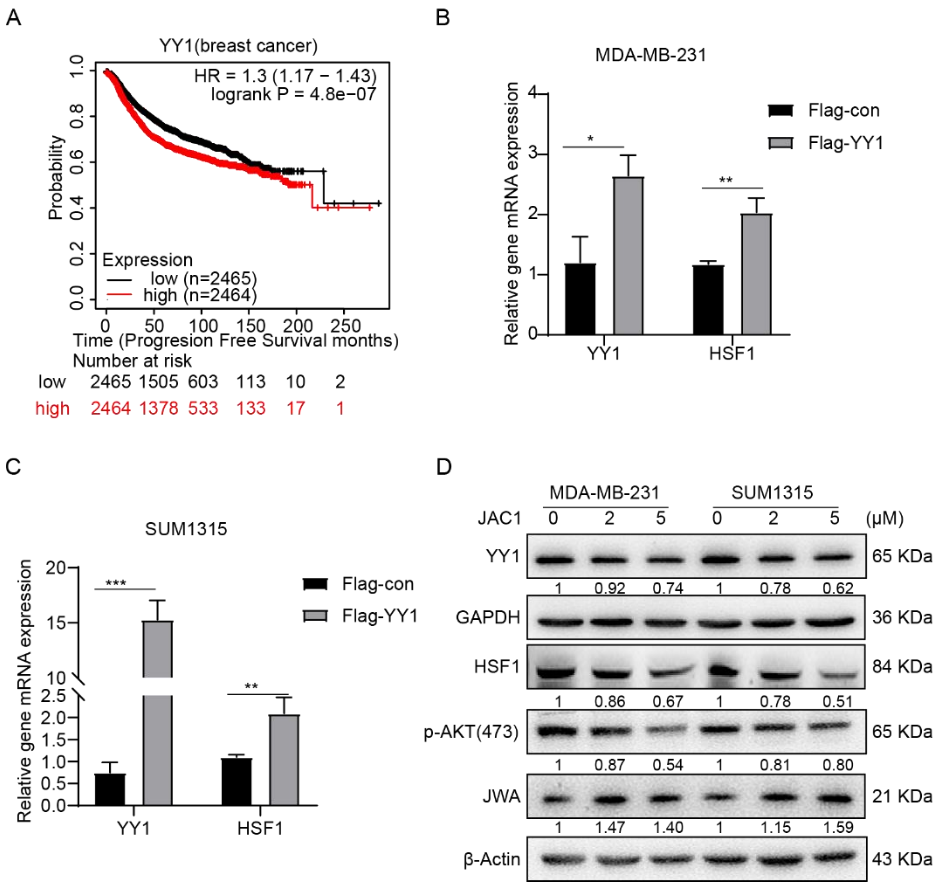

Supplementary Fig. 5 JAC1 regulates the expression of YY1 downstream genes.

A Kaplan–Meier curves depicted survival according to the expression of YY1 in breast cancer cohort from TCGA database. *P* values were calculated with the log-rank test. **B-C** YY1 negatively regulated the mRNA expression of HSF1 in both MDA-MB-231(**B**) and SUM1315(**C**) cells (n=3). **D** The expressions of YY1, HSF1 and p-AKT (473) were detected by Western blot in in both MDA-MB-231 and SUM1315 cells after treated by indicated doses of JAC1 for 24 h. * $P < 0.05$, ** $P < 0.01$, *** $P<0.001$.

Supplemental Tables

Supplemental Table1 : Information for antibodies.

Antibody	Vendor	Catalogue number	Application	Dilution
YY1	proteintech	22156-1-AP	WB	1:2000
			IP	1:200
			IF	1:200
CSDE1	Affinity	DF12920	WB	1:2000
p53	Affinity	AF0879	WB	1:2000
ZFX	SANTA	Sc-271984	WB	1:100
STAT1	Affinity	AF6299	WB	1:1000
LARP7	Affinity	DF12208	WB	1:2000
p38	ABclonal	A4771	WB	1:2000
p-p38(Thr180/Thr182)	Affinity	AF4001	WB	1:2000
Bcl2	proteintech	12789-1-AP	WB	1:4000
Bax	proteintech	50599-2-lg	WB	1:10000
CyclinD1	proteintech	26939-1-AP	WB	1:2000
p21	proteintech	10355-1-AP	WB	1:1000
HSF1	proteintech	16107-1-AP	WB	1:1000
Cleaved-caspase3	proteintech	19677-1-AP	WB	1:2000
Ub	CST	3936T	WB	1:1000
JWA	Lab homemade		WB	1:100
PCNA	CST	#13110	WB	1;1000
p-ERK	CST	#4370	WB	1:1000
p-JNK	Affinity	AF3318	WB	1:2000
GAPDH	Beyotime	AF5009	WB	1:1000
β-actin	Beyotime	AA128	WB	1:1000
Tubulin	Beyotime	AF1216	WB	1:1000
Lamin B1	proteintech	12987-AP	WB	1:10000

Supplemental Table2 : The serum biochemical indexes (15 parameters) in control and JAC1-treated mice.

Analysis	Groups		[Unit]
	Control（n=8）	100 mg/kg JAC1（n=8）	
[TP]	29.41±2.016	31.18±3.177	g/L
[ALB]	23.1±2.186	25.08±3.226	g/L
[TBIL]	0.6125±0.1959	0.6±0.1512	μmol/L
[ALP]	90.25±3.67	90.38±22.17	U/L
[GGT]	3.5±1.852	2.875±1.885	U/L
[GLU]	9.638±2.157	7.8±1.721	mmol/L
[BUN]	7.588±1.38	7.988±0.7453	mmol/L
[CREA]	15.75±2.712	15.5±0.9258	μmol/L
[UA]	153±25.35	163.6±39.99	μmol/L
[Ca]	2.214±0.09486	2.259±0.1876	mmol/L
[P]	2.98±0.4458	2.4±0.5189	mmol/l
[CHOl]	2.179±0.3679	2.181±0.325	mmol/L
[TG]	0.8663±0.1761	0.9838±0.1769	mmol/L
[HDL-C]	1.29±0.1917	1.409±0.2088	mmol/L
[LDL-C]	0.215±0.07982	0.1238±0.0447	mmol/L
