# Supplementary material for: JAC1 targets YY1 mediated JWA/p38 MAPK signaling to inhibit proliferation and induce apoptosis in TNBC
Source: Cell Death Discov. 2022 Apr 5;8:169. doi: 10.1038/s41420-022-00992-9 (PMC8983694; doi:10.1038/s41420-022-00992-9)

Original Western blot data

Fig.1H

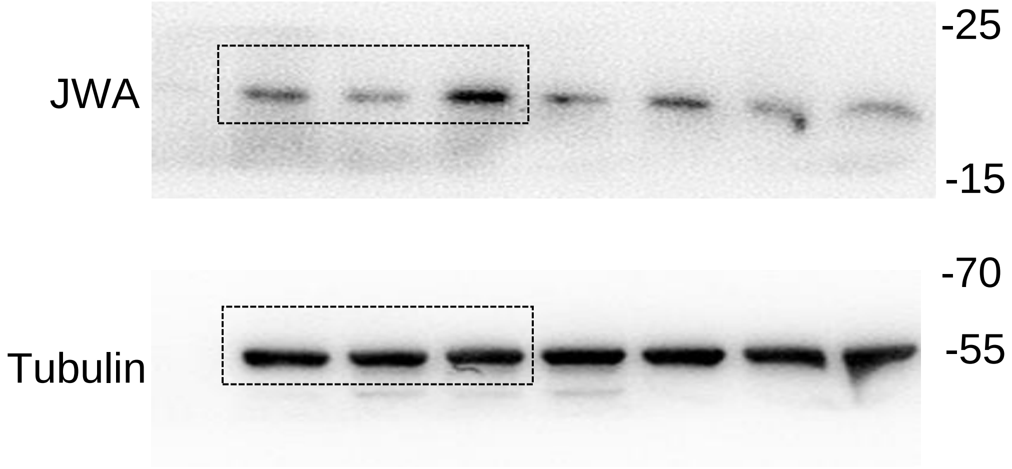

Fig. 2I

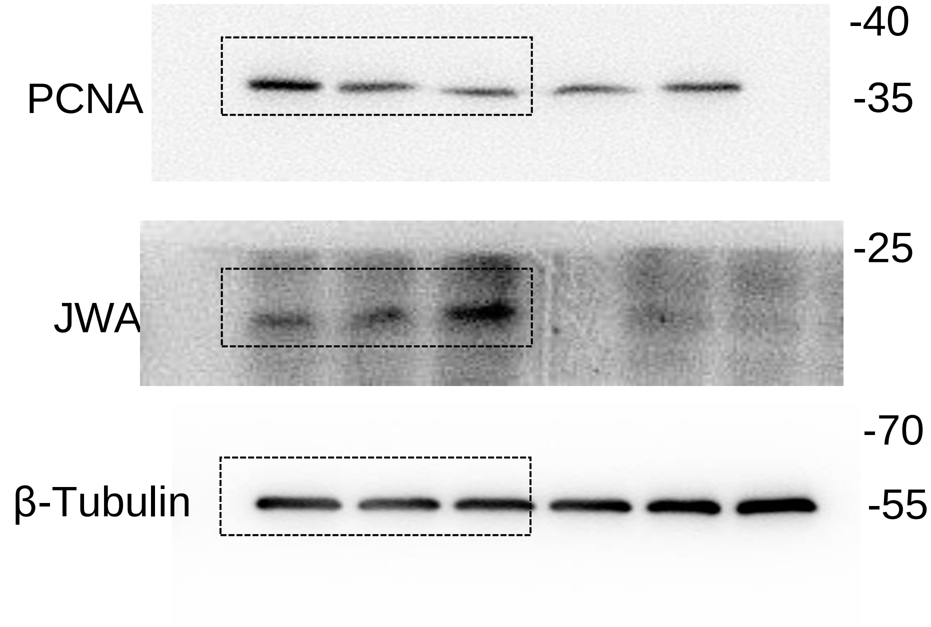

Fig. 2H

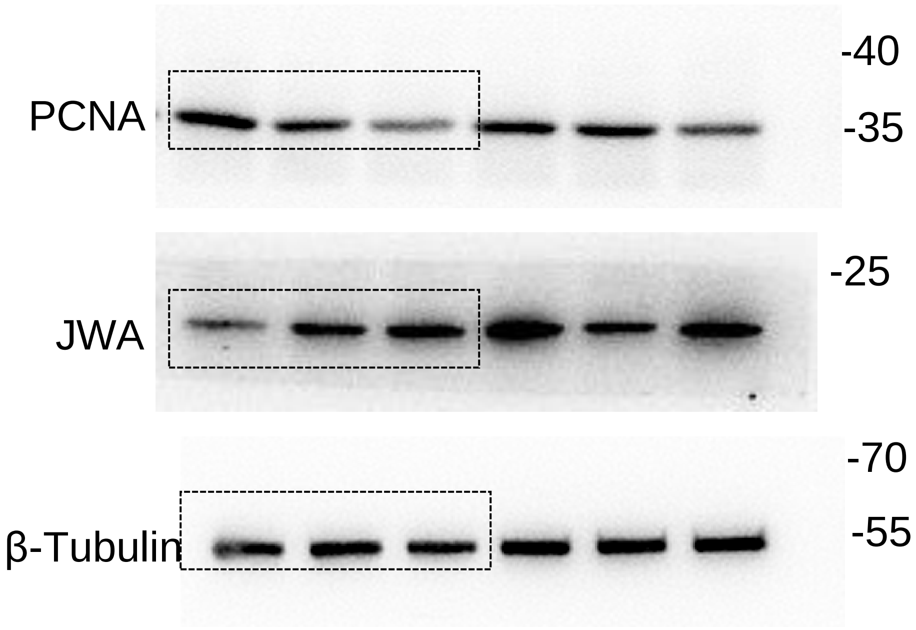

Fig. 3E（left）

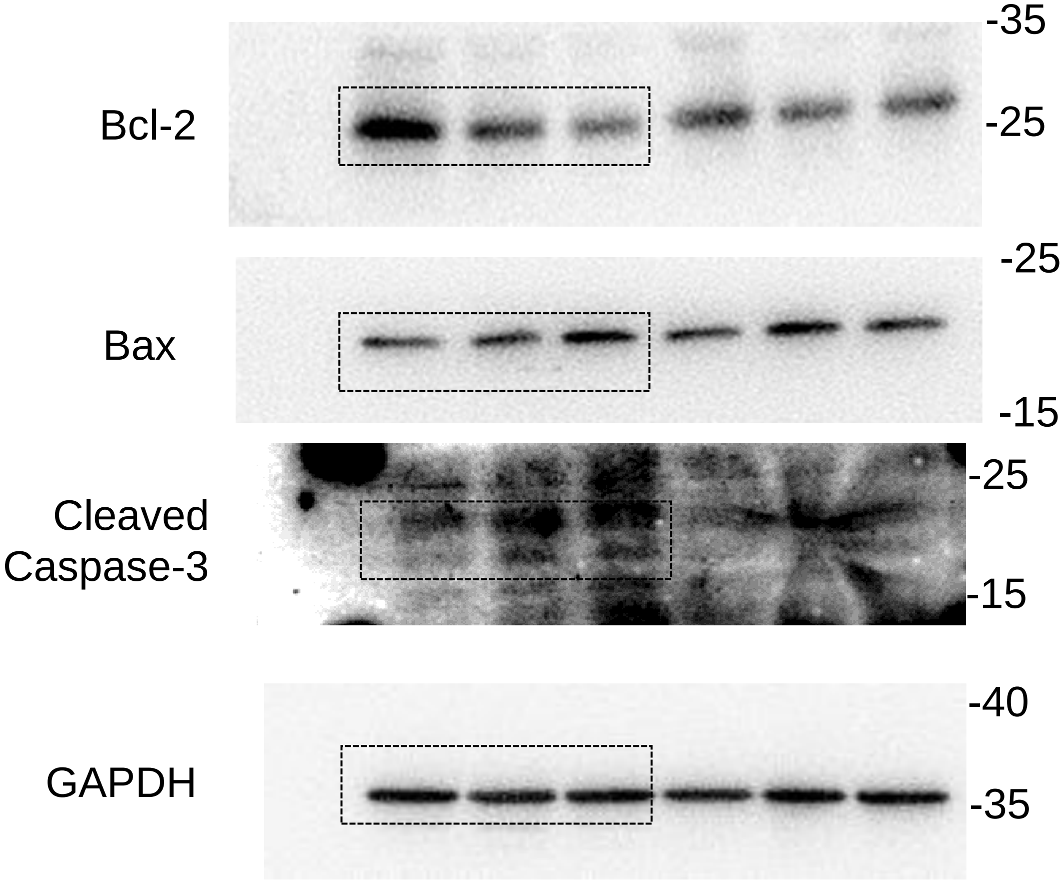

Fig. 3E（right）

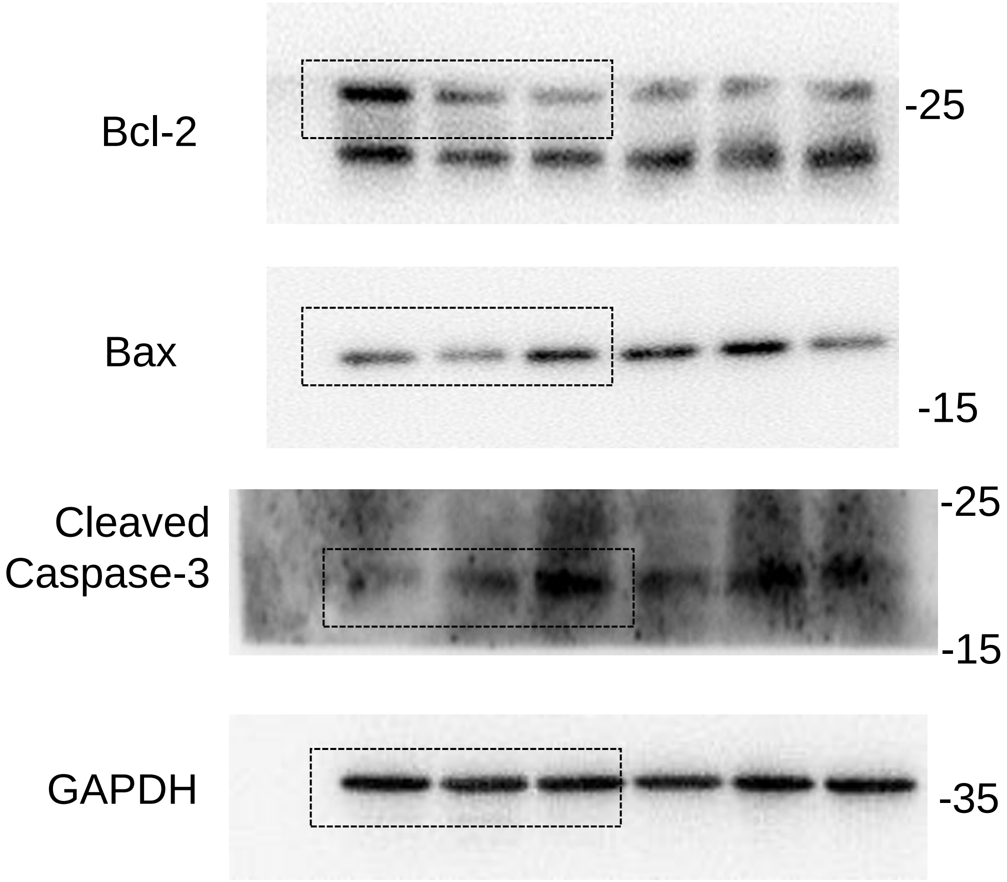

Fig. 3J （left）

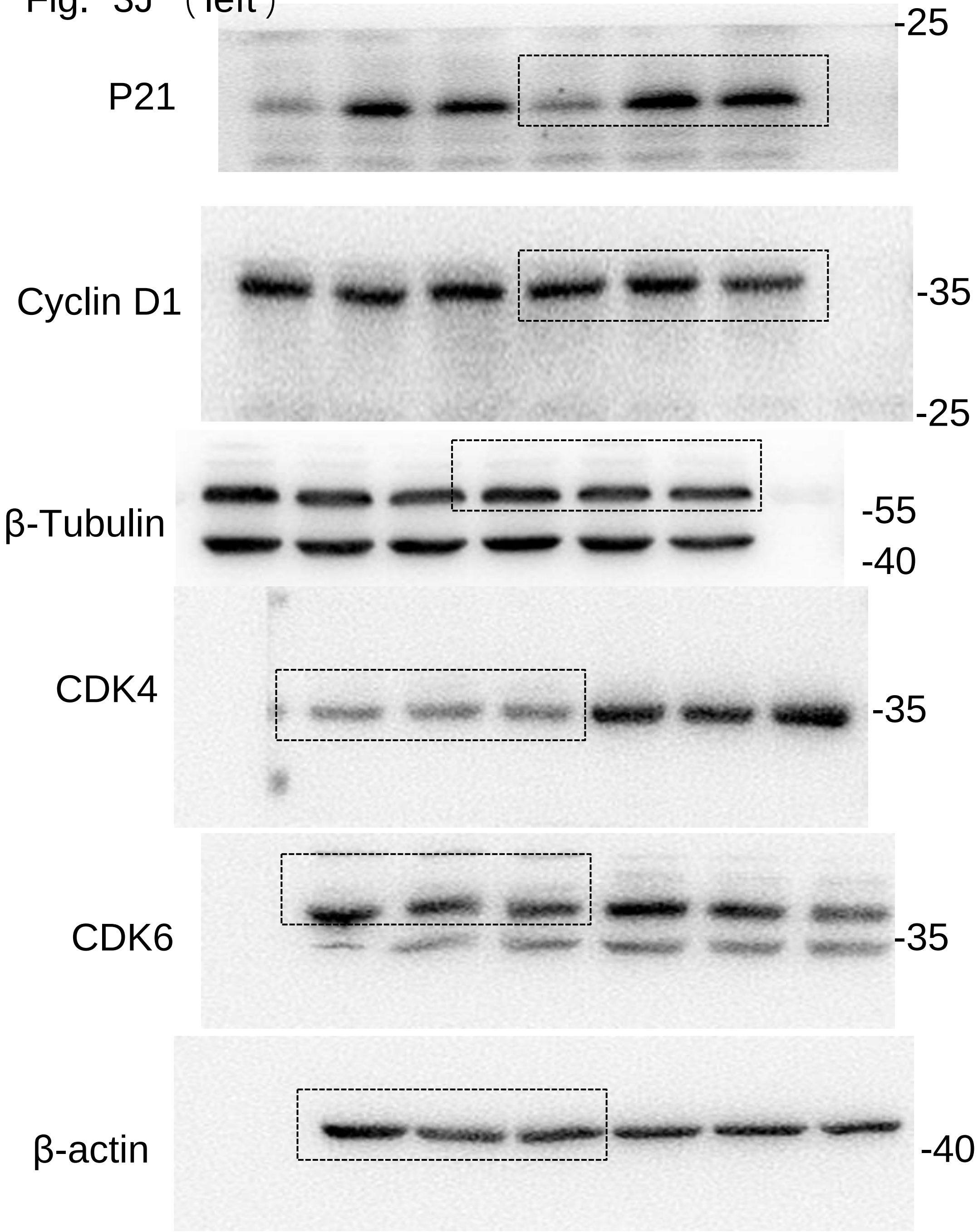

Fig. 3J （right）

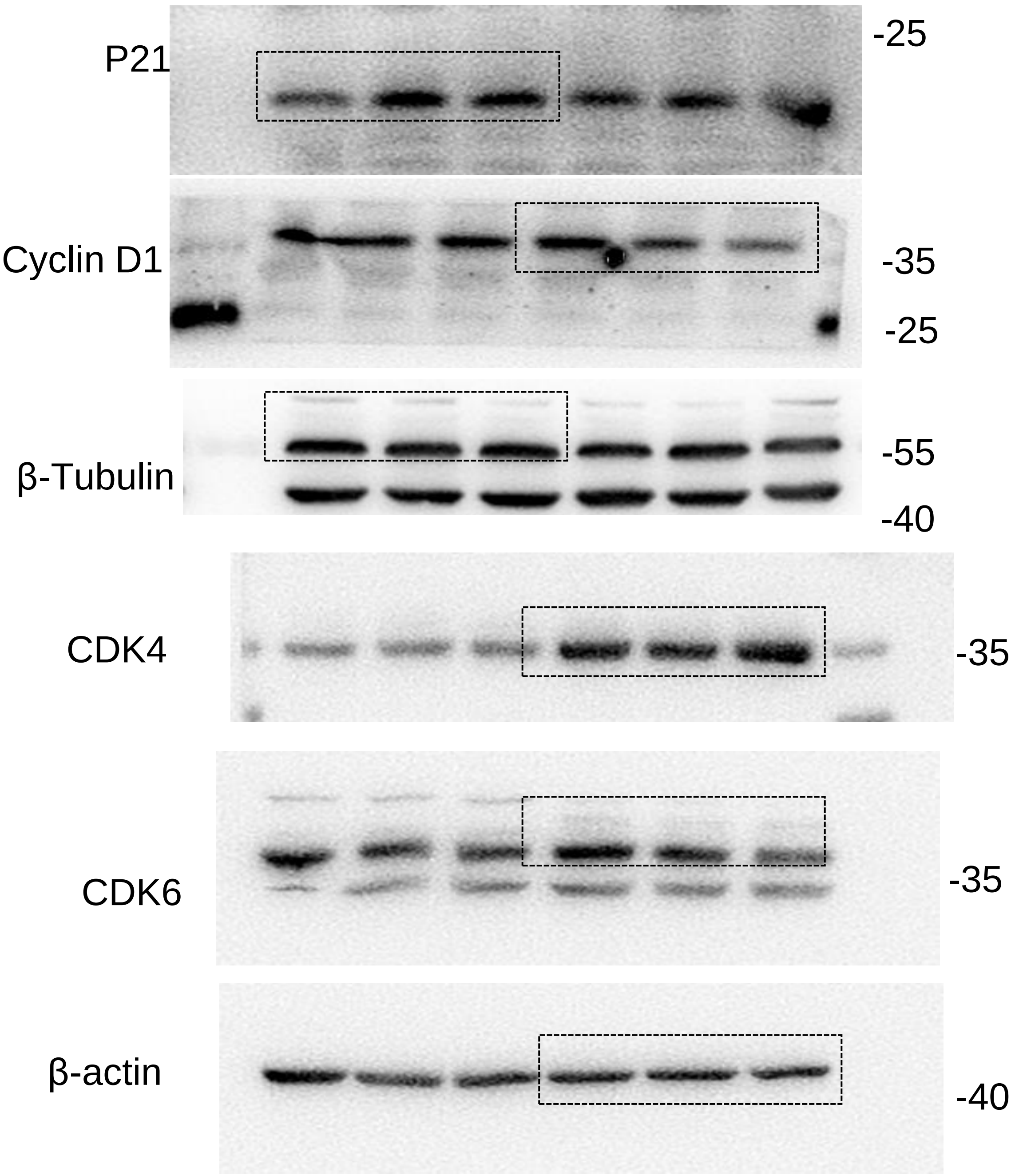

Fig. 4E

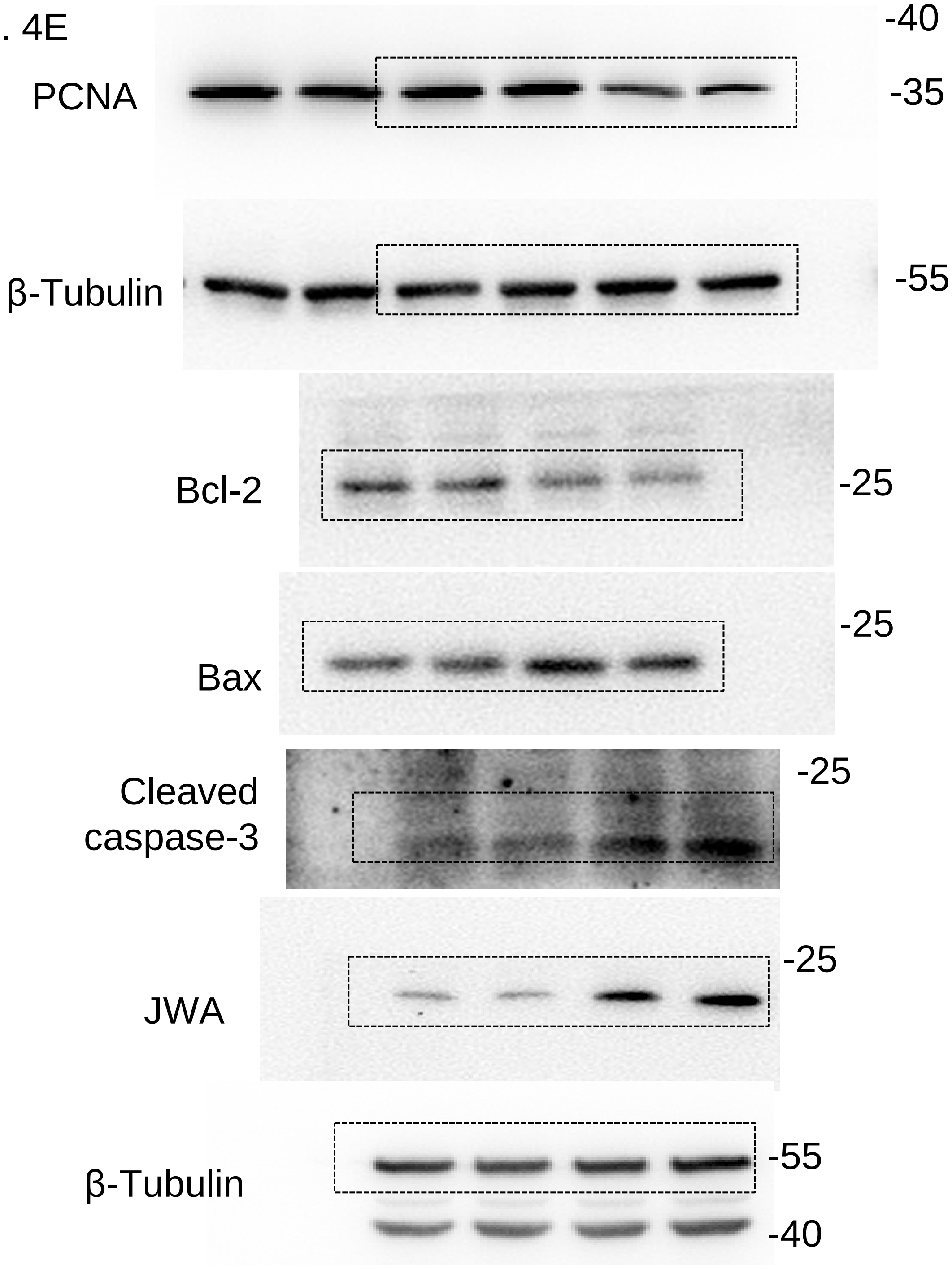
PCNA
β-Tubulin
Bcl-2
Bax
Cleaved caspase-3
JWA
β-Tubulin
-40
-35
-55
-25
-25
-25
-25
-55
-40

Fig. 4E

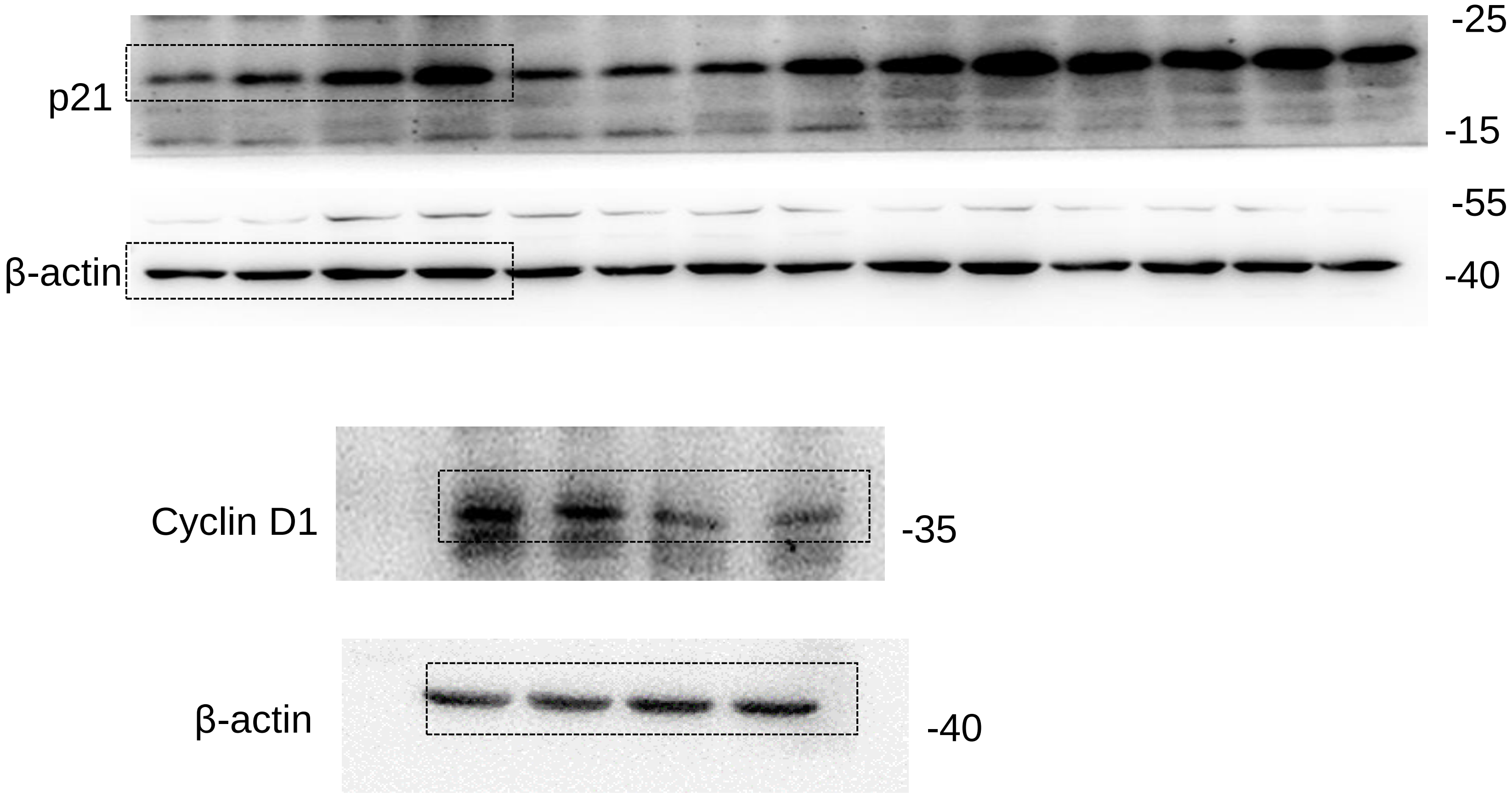

Fig. 5A

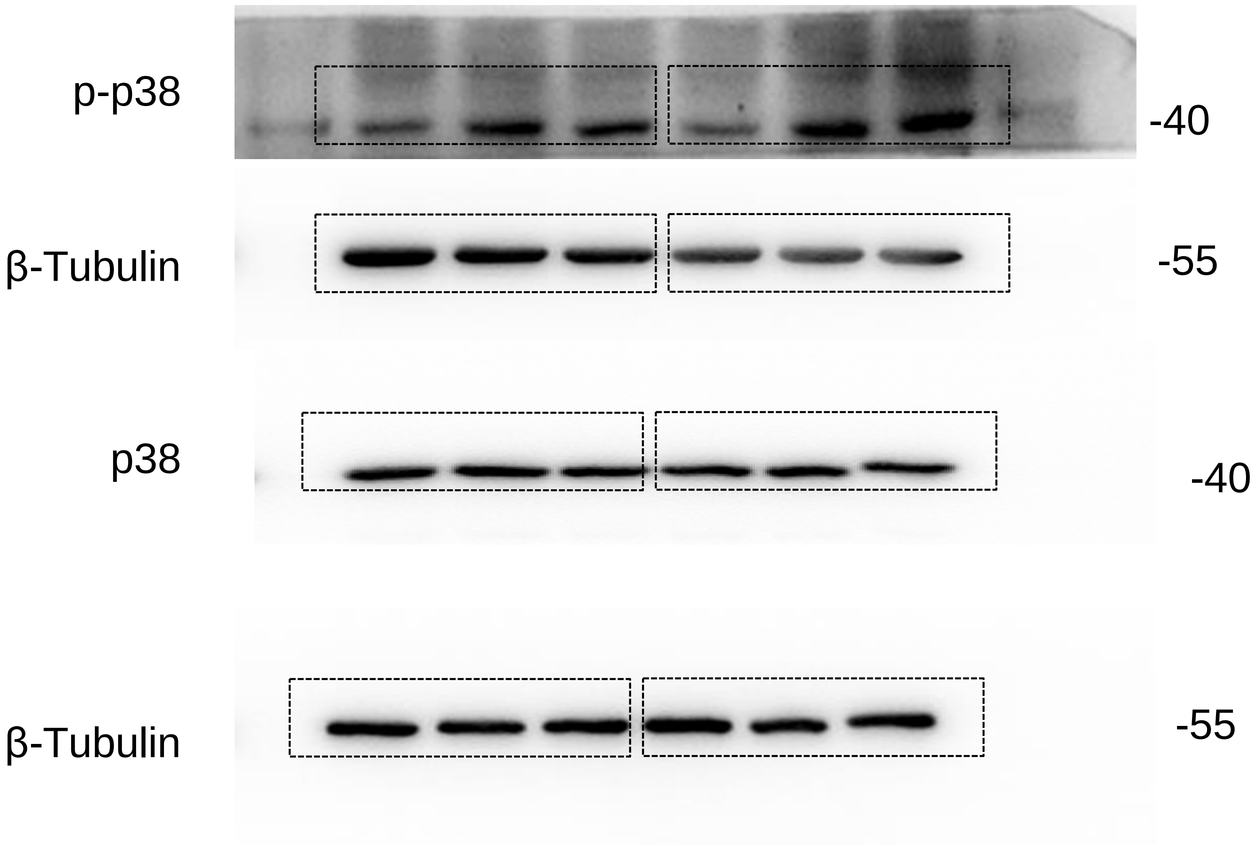

Fig. 5I(left)

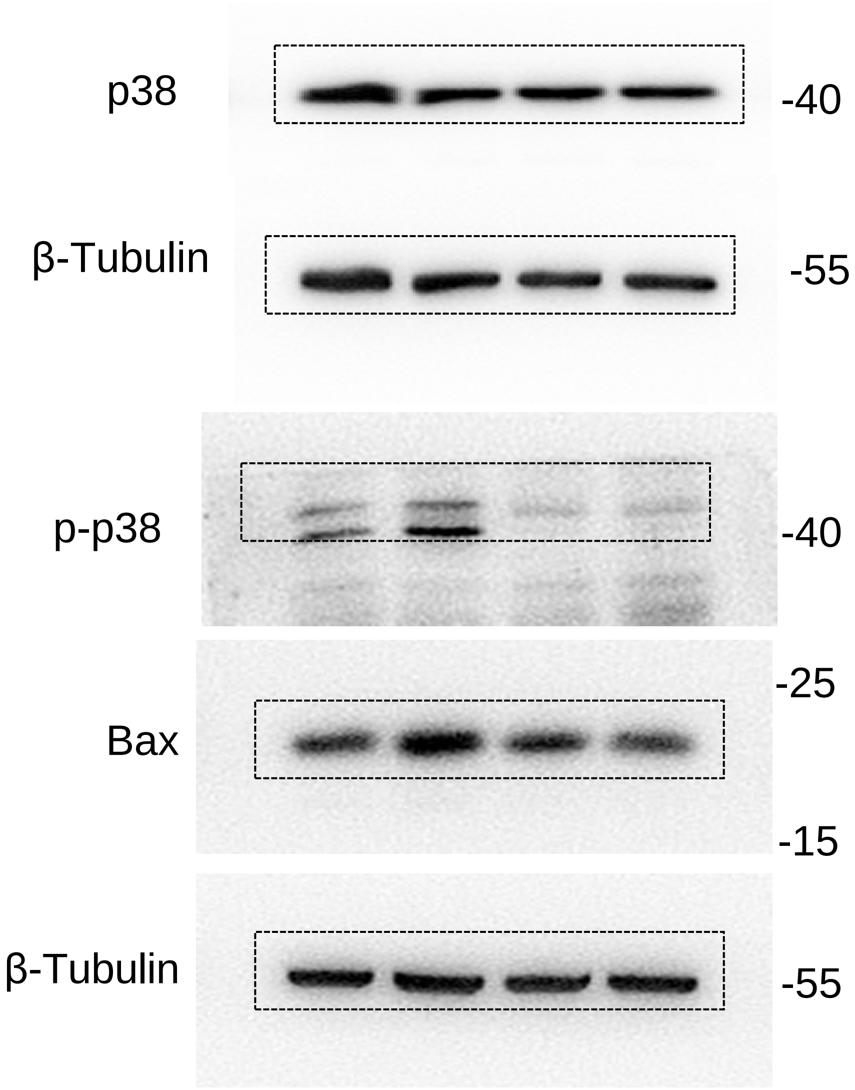

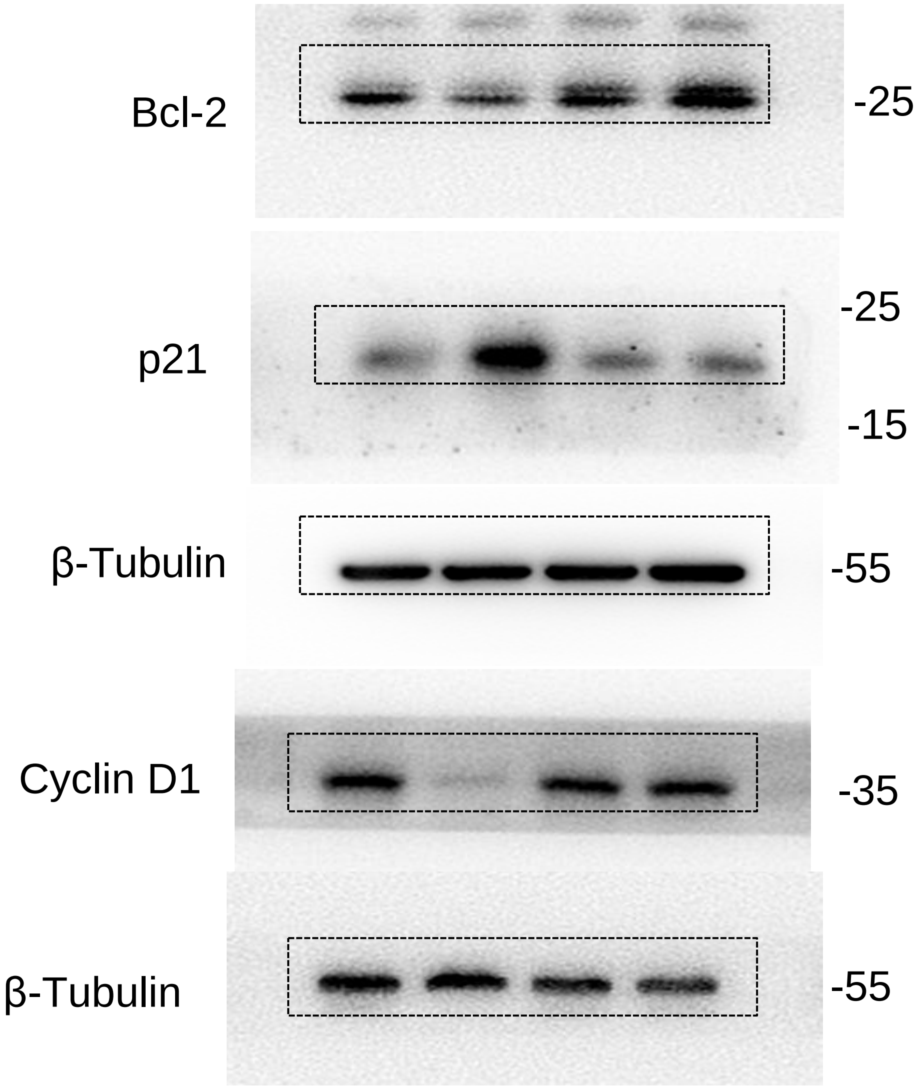

Bcl-2
-25
p21
-25
-15
β-Tubulin
-55
Cyclin D1
-35
β-Tubulin
-55

Fig. 5I(right)

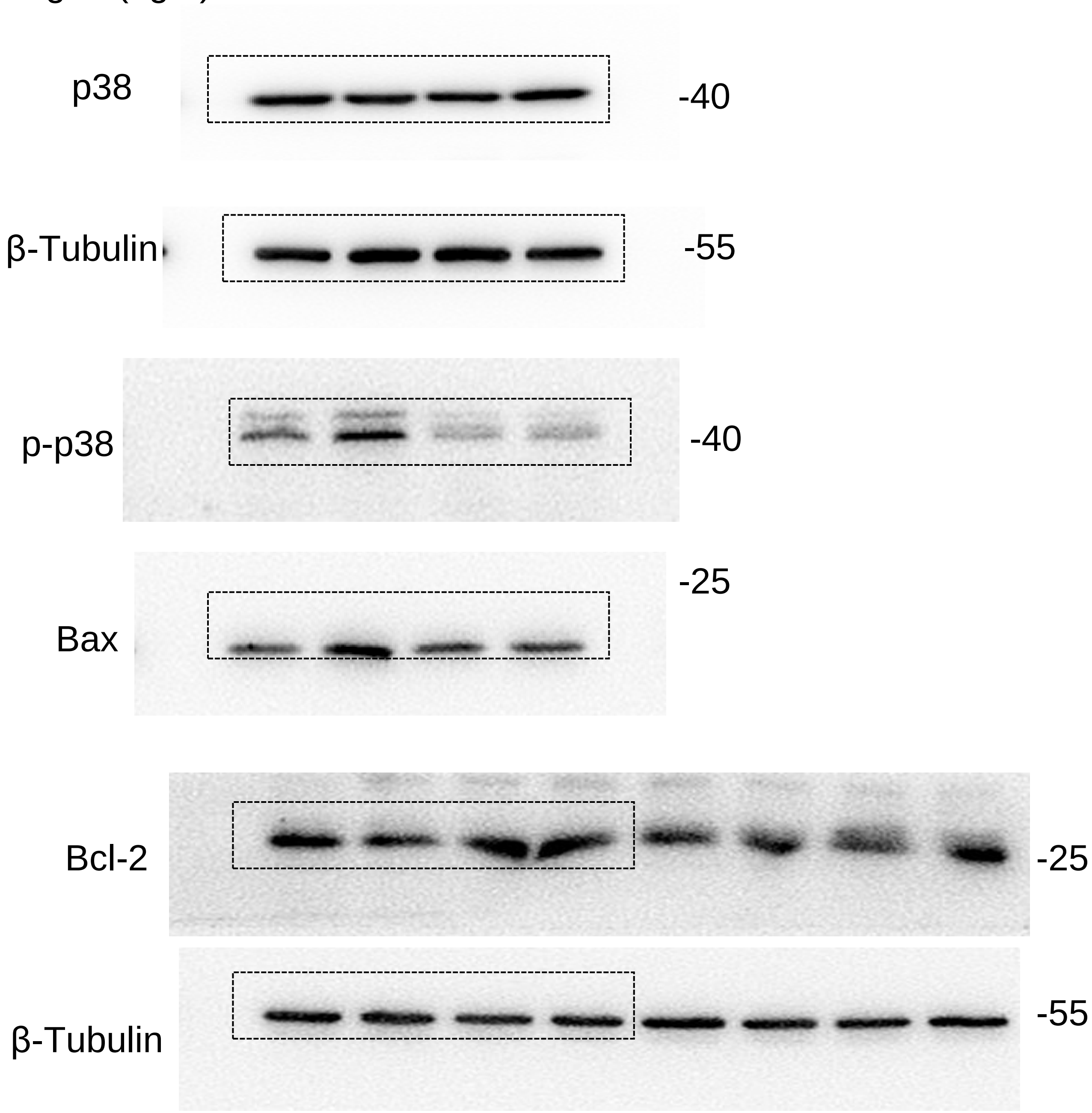

Fig. 5I(right)

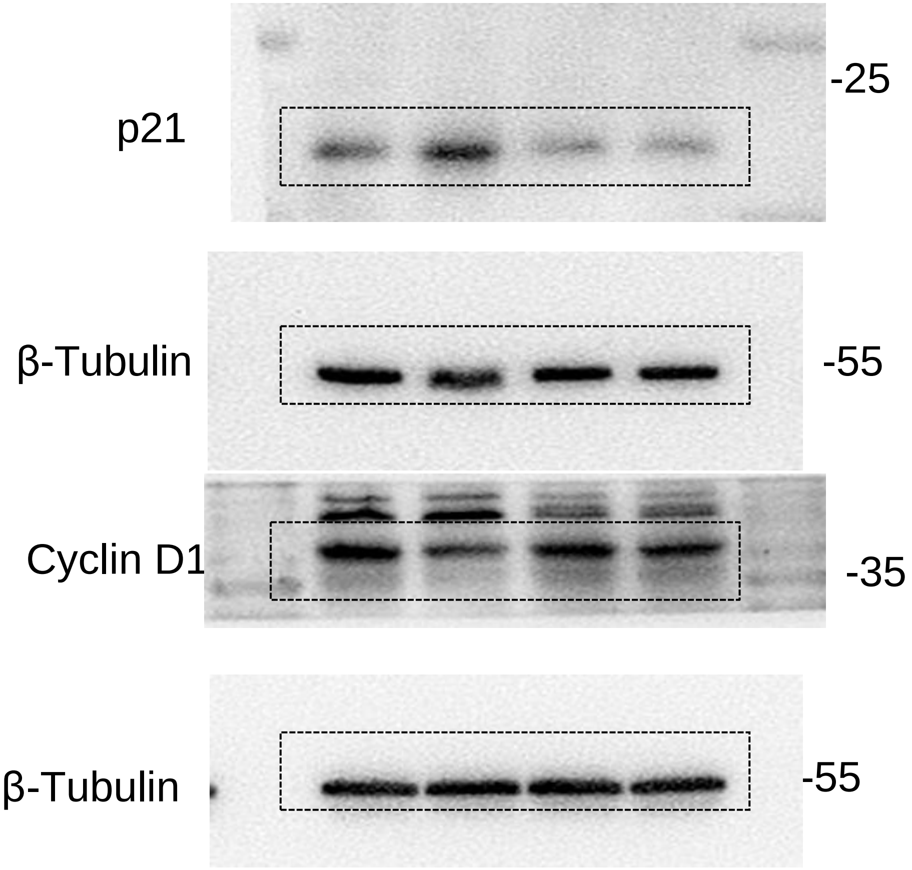

Fig. 6A

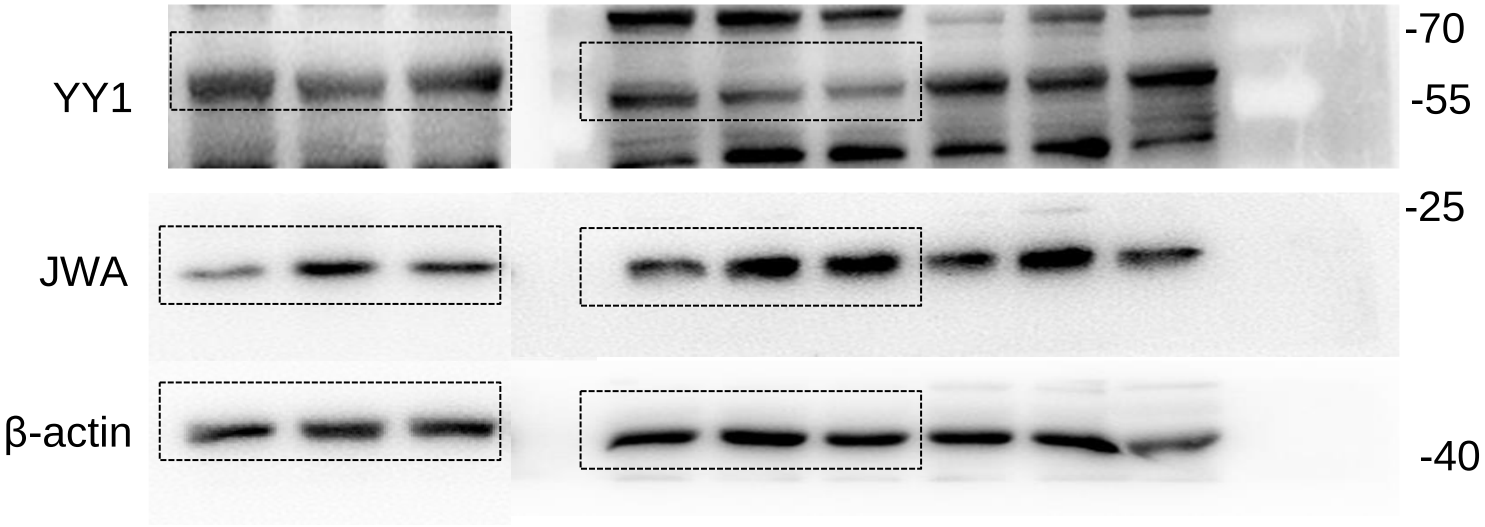

Fig.6B

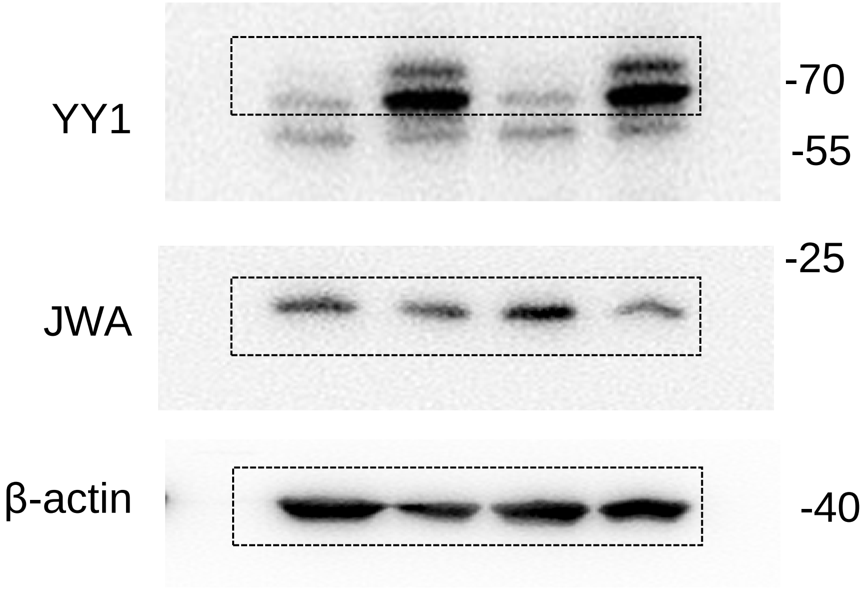

Fig. 6C

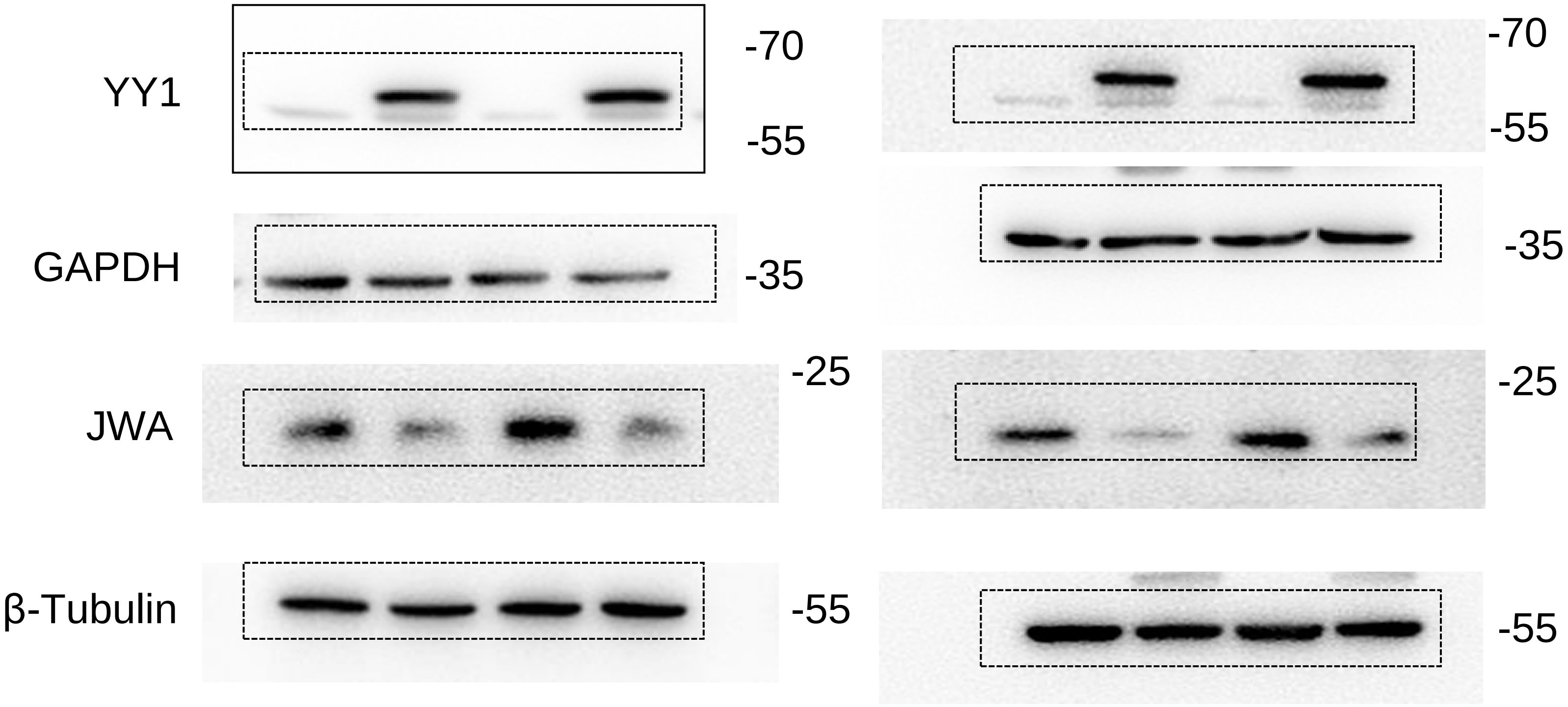

Fig.7A

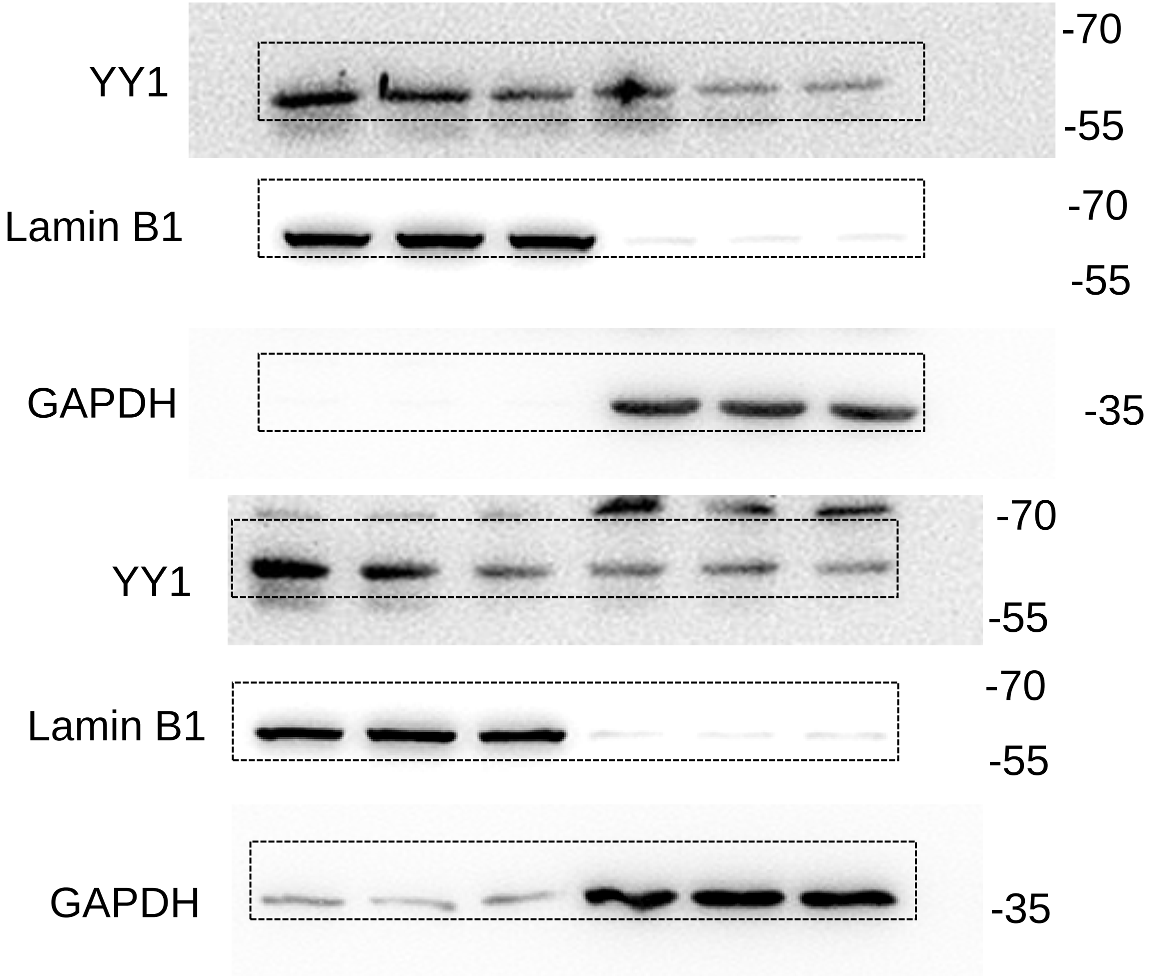

YY1
-70
-55
Lamin B1
-70
-55
GAPDH
-35
YY1
-70
-55
Lamin B1
-70
-55
GAPDH
-35

Fig. 7B

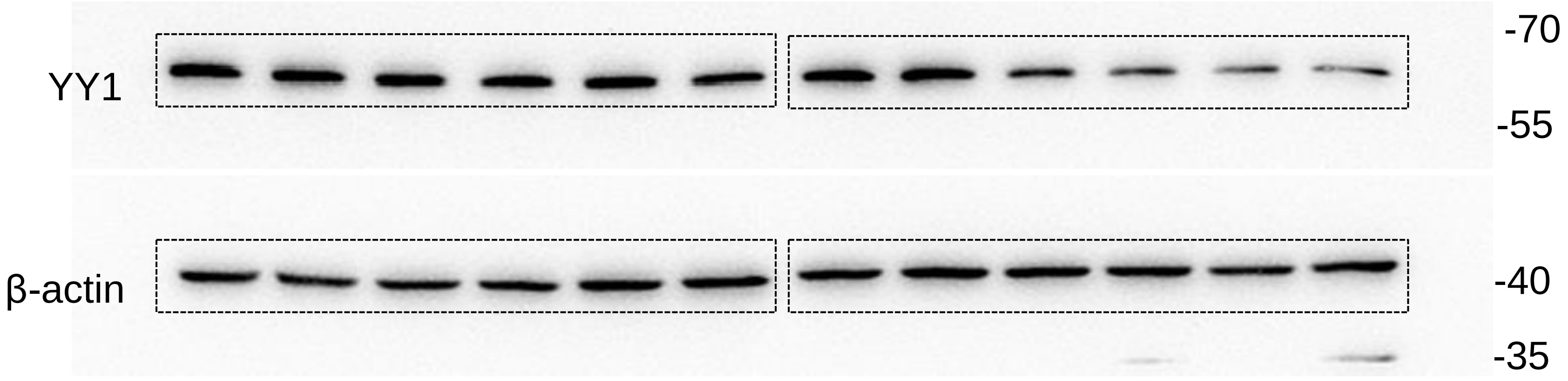

YY1
-70
-55
β-actin
-40
-35

Fig. 7E

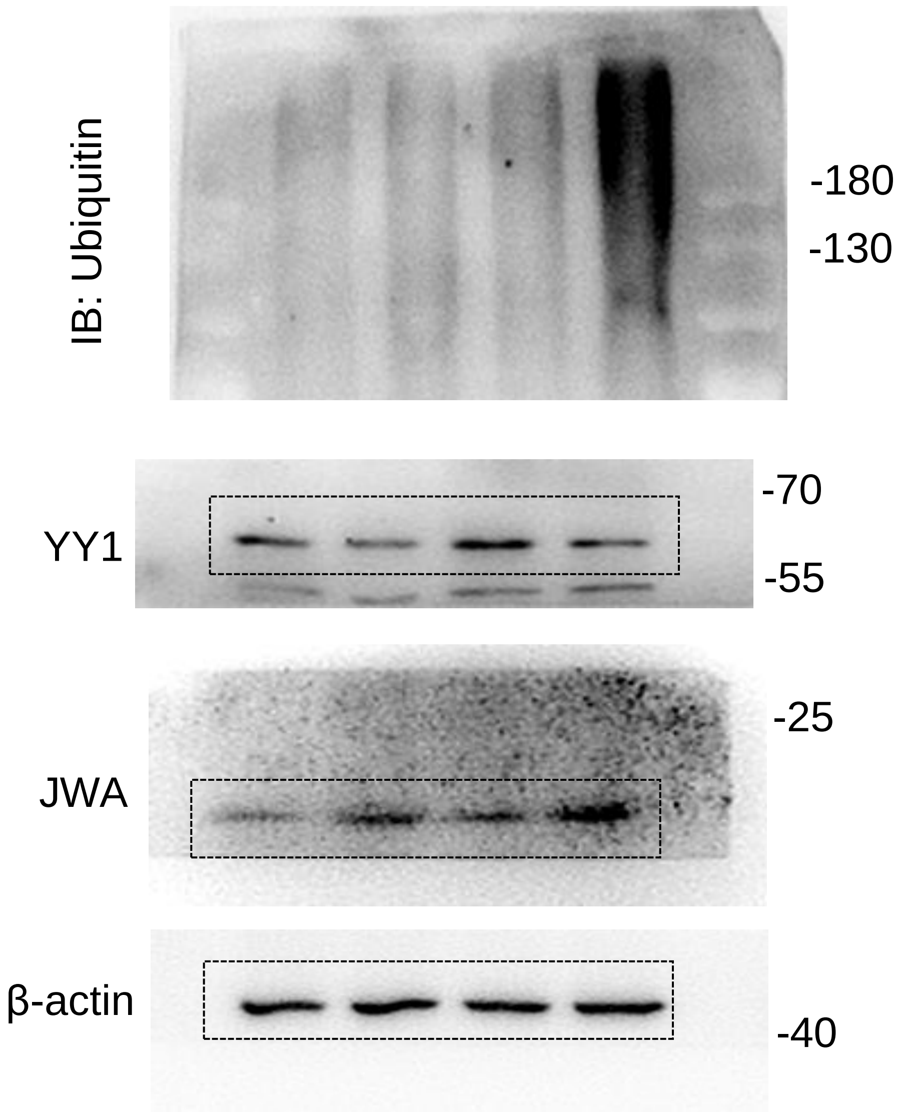
IB: Ubiquitin
-180
-130
YY1
-70
-55
JWA
-25
β-actin
-40

Fig. 7F

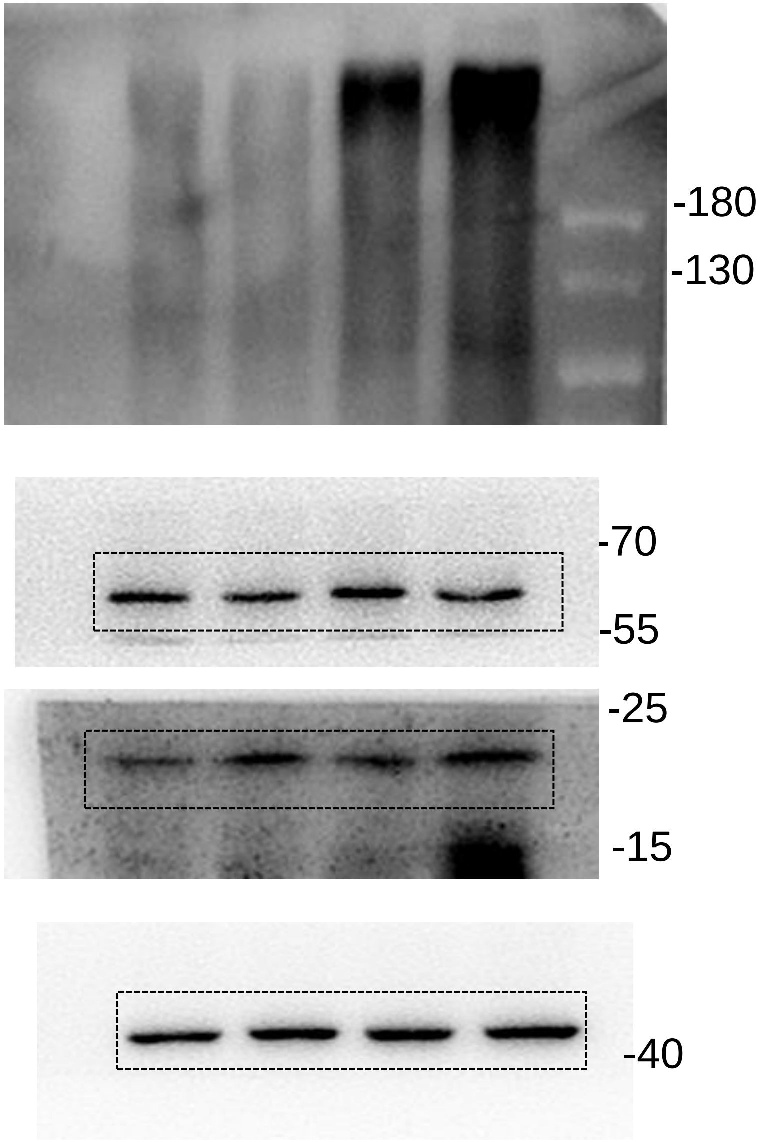
-180
-130
-70
-55
-25
-15
-40

Fig. S 2A

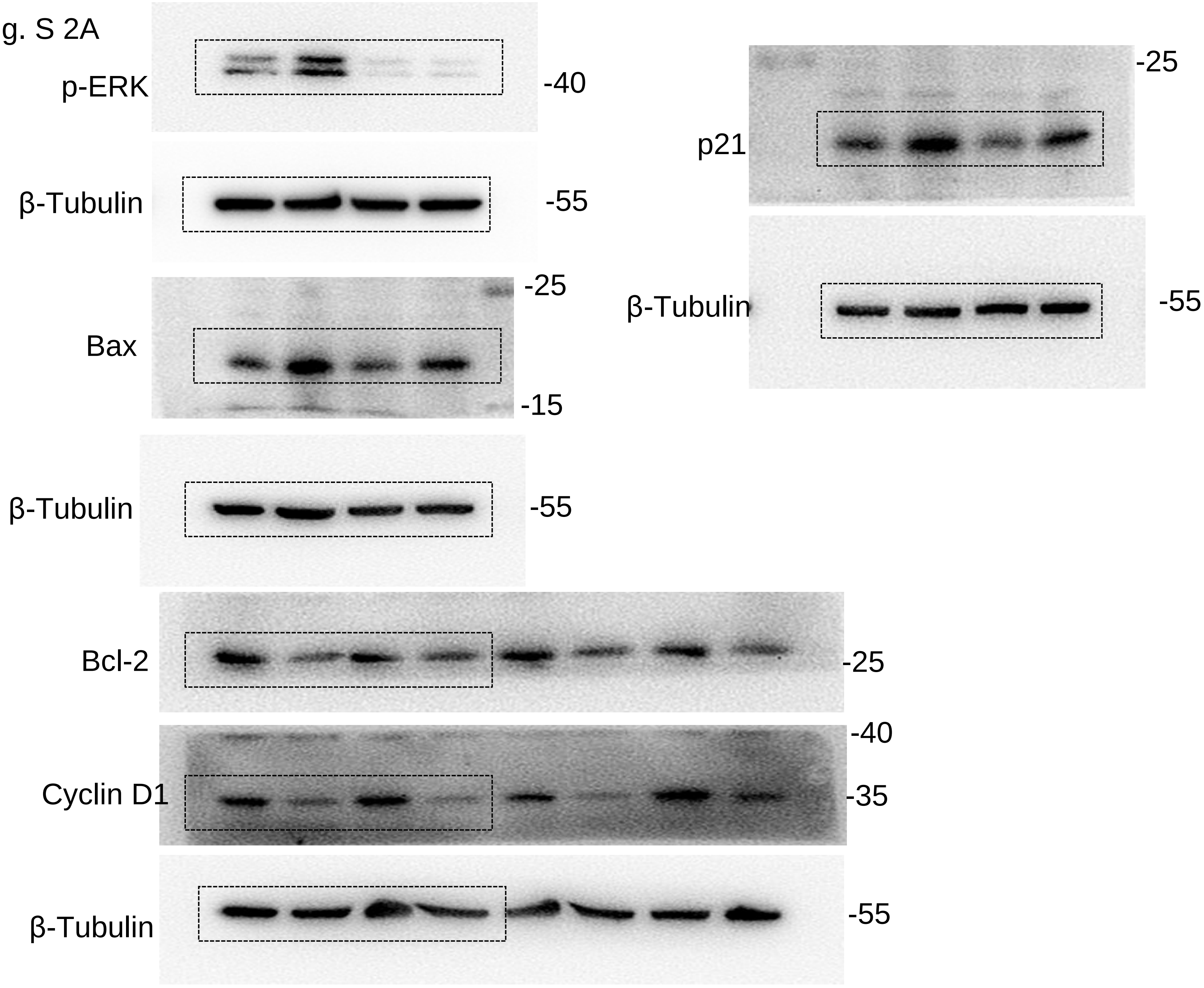
p-ERK
-40
β-Tubulin
-55
Bax
-25
-15
β-Tubulin
-55
Bcl-2
-25
Cyclin D1
-40
-35
β-Tubulin
-55
p21
-25
β-Tubulin
-55

Fig.S2B

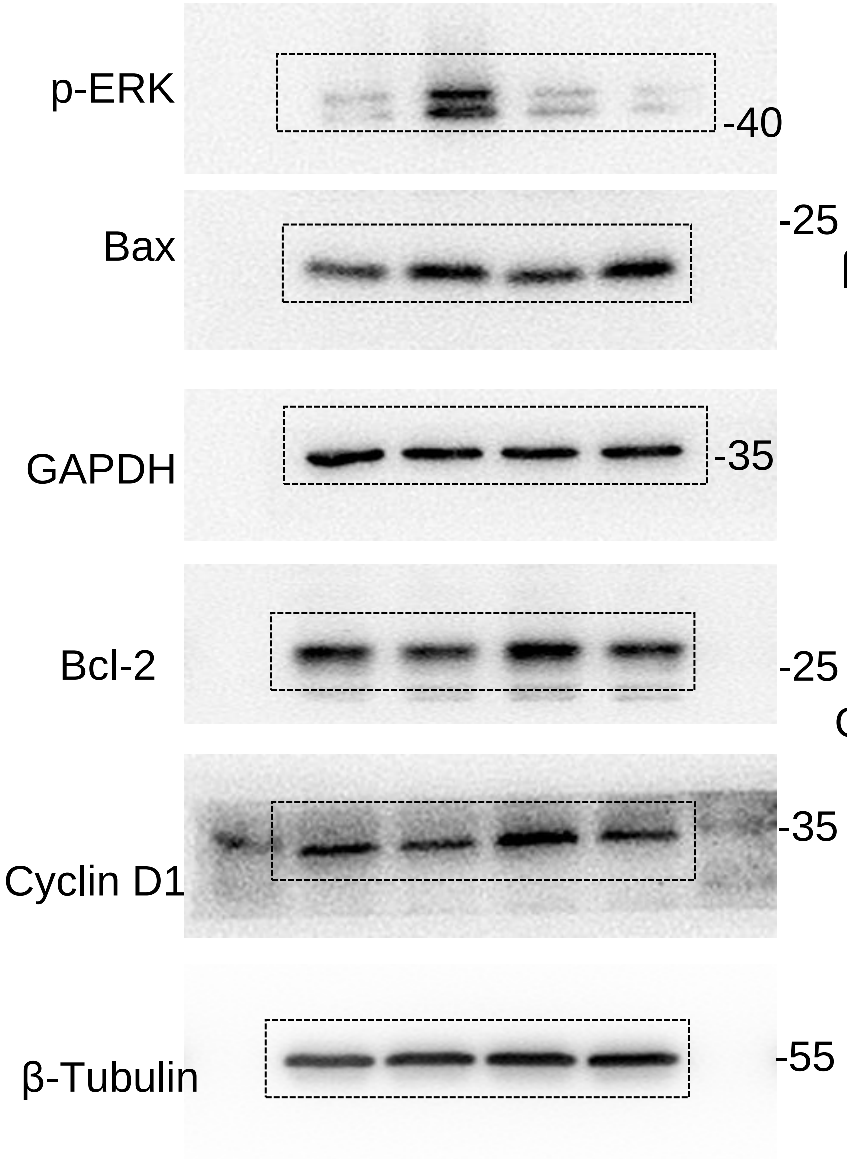

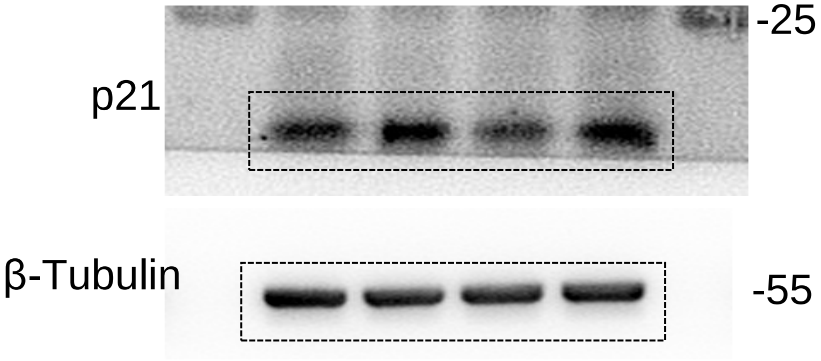

Fig.S2C

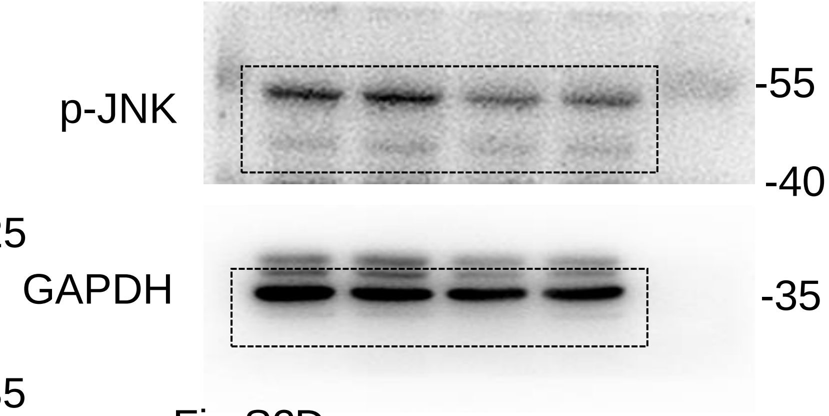

Fig.S2D

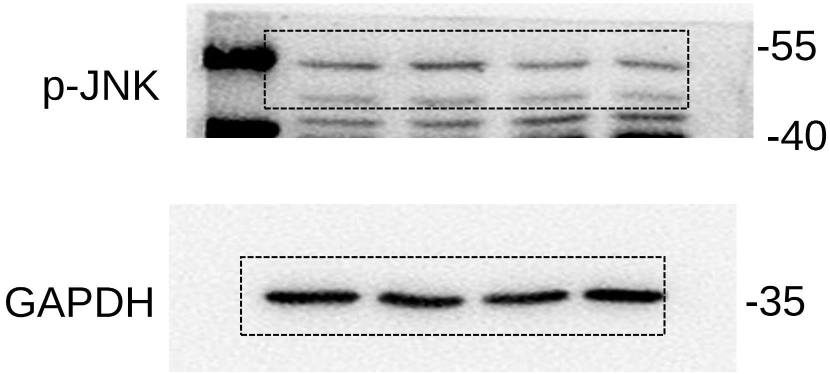

Fig. S 3A

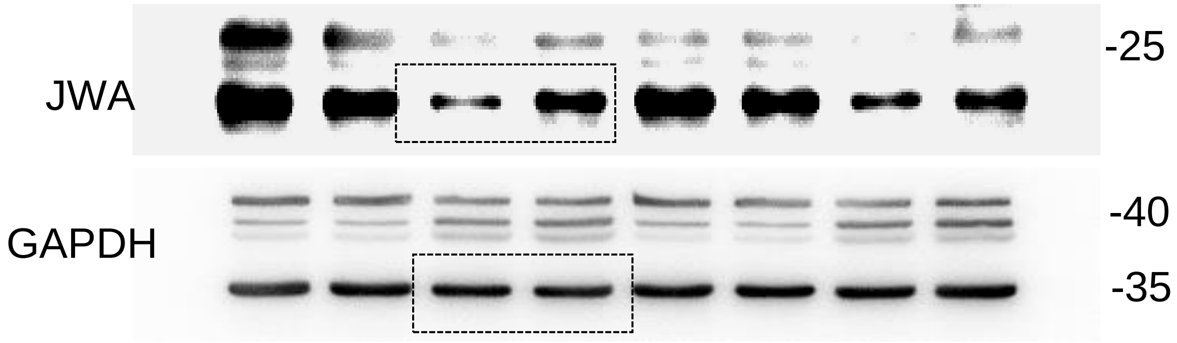

Fig. S 3C

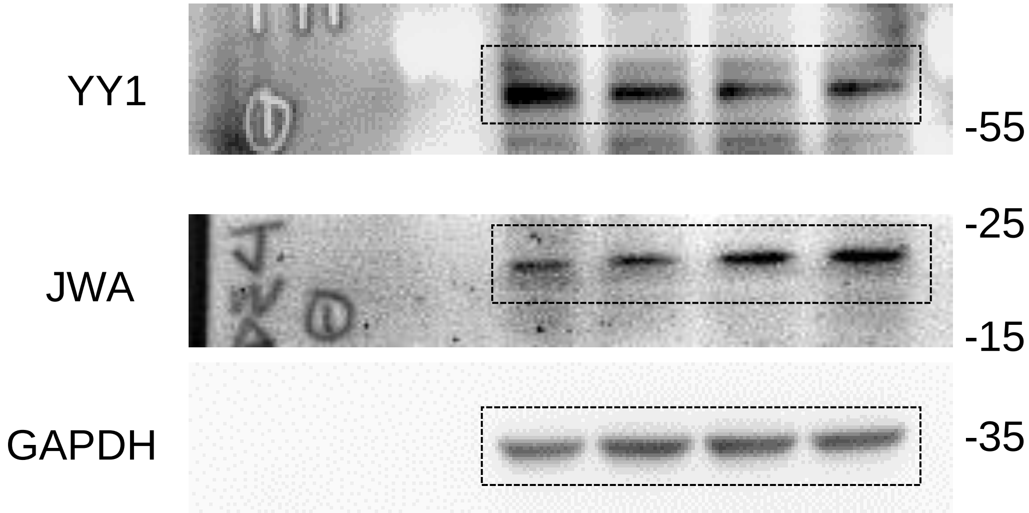

Fig.S3D

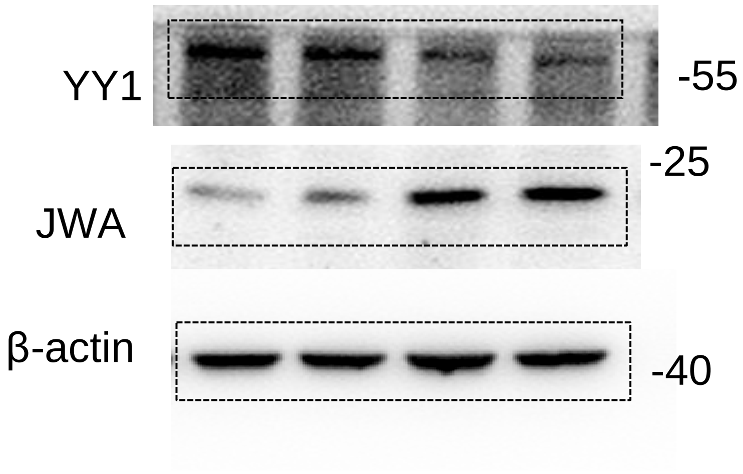

Fig.S3F

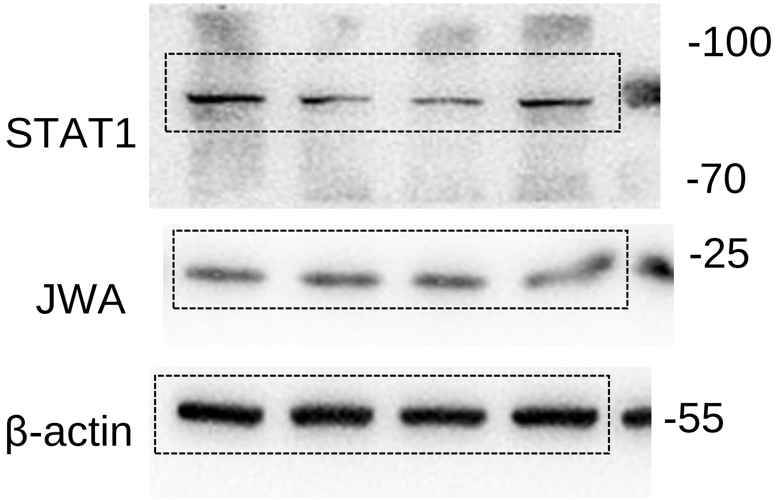

Fig.S3E

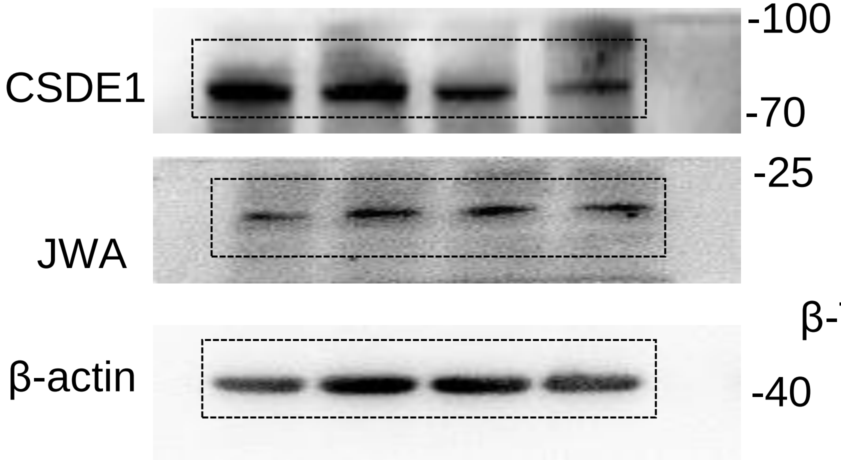

Fig.S3G

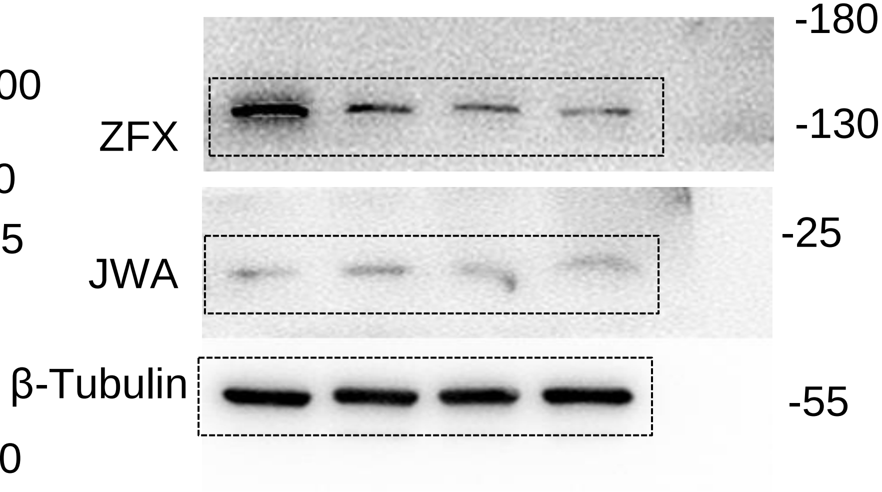

Fig. S3H

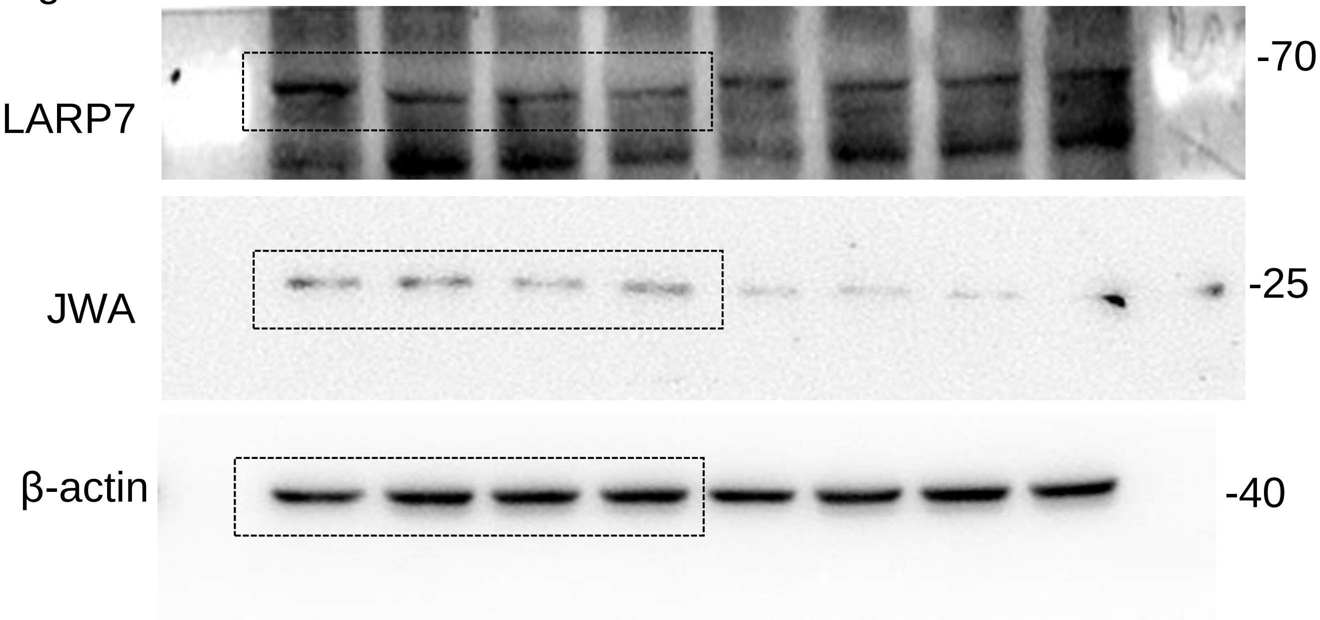

Fig. S 3I

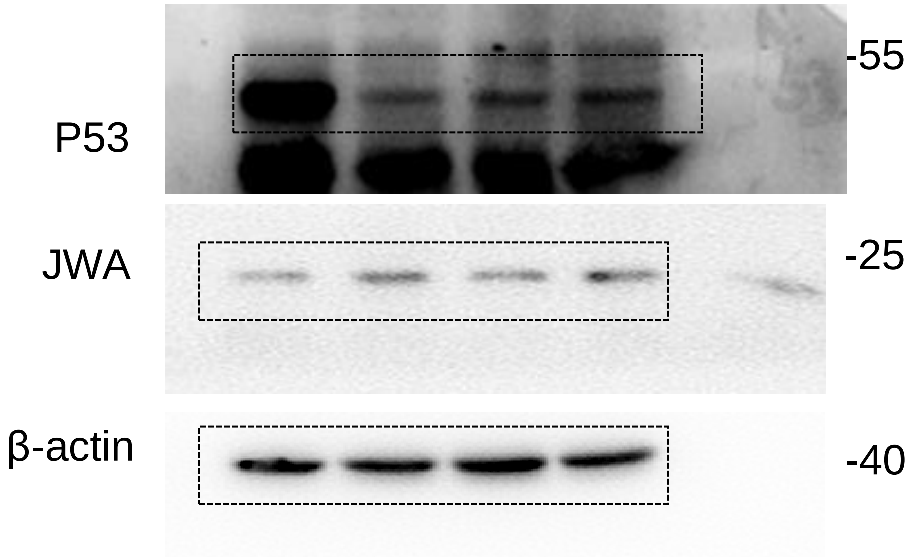

Fig. S 4B

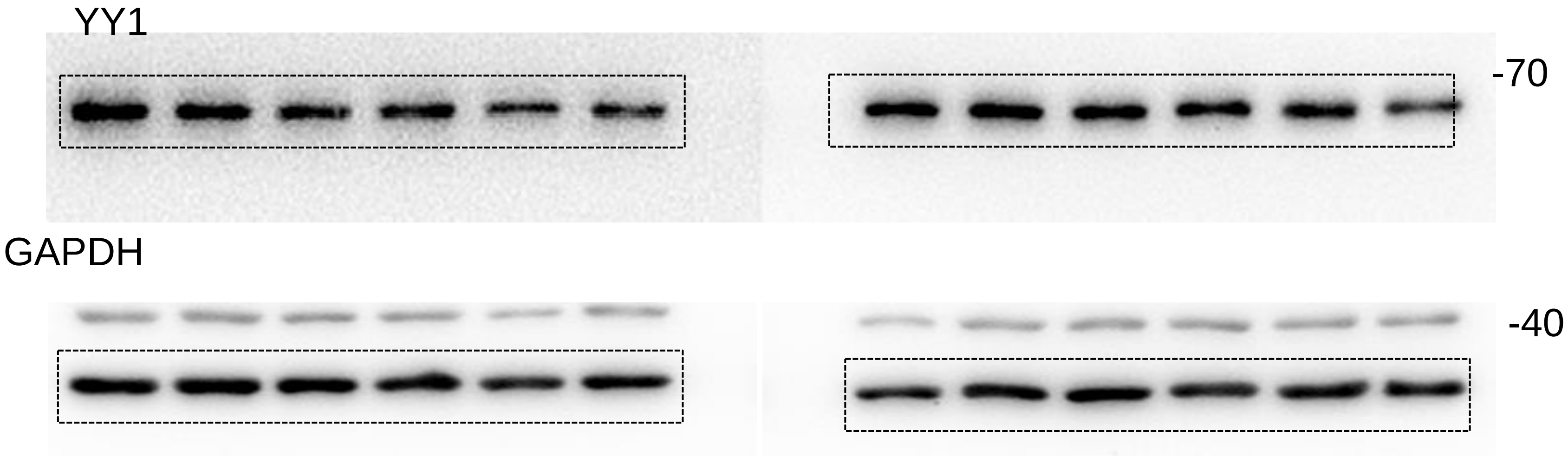

Fig. S 5D

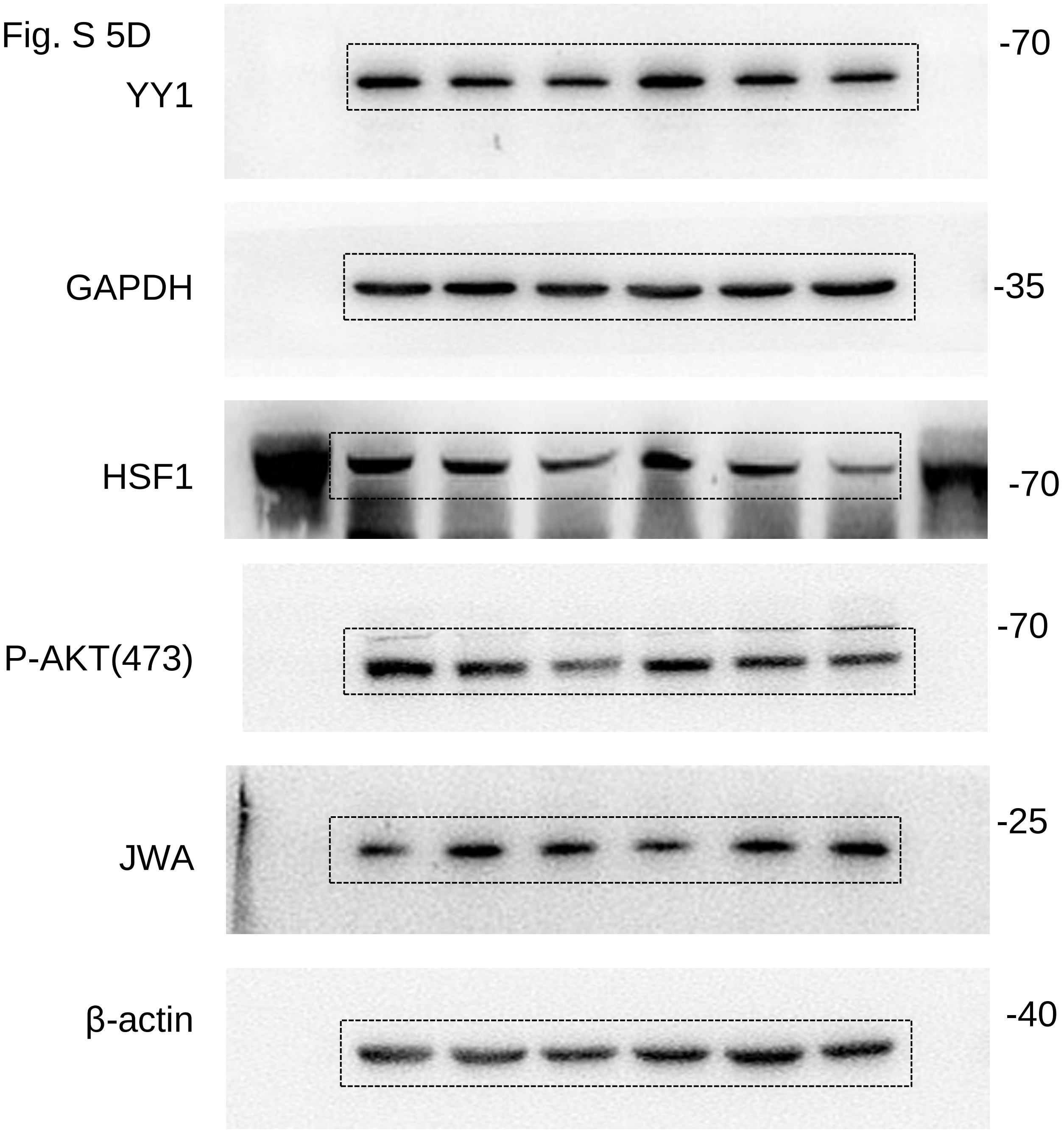

Supplement: Supplementary file 2 — Original Data File [file 41420_2022_992_MOESM2_ESM.pdf]
